# Identifying novel regulatory effects for clinically relevant genes through the study of the Greek population

**DOI:** 10.1186/s12864-023-09532-w

**Published:** 2023-08-05

**Authors:** Konstantinos Rouskas, Efthymia A. Katsareli, Charalampia Amerikanou, Alexandros C. Dimopoulos, Stavros Glentis, Alexandra Kalantzi, Anargyros Skoulakis, Nikolaos Panousis, Halit Ongen, Deborah Bielser, Alexandra Planchon, Luciana Romano, Vaggelis Harokopos, Martin Reczko, Panagiotis Moulos, Ioannis Griniatsos, Theodoros Diamantis, Emmanouil T. Dermitzakis, Jiannis Ragoussis, George Dedoussis, Antigone S. Dimas

**Affiliations:** 1 Institute for Bioinnovation, Biomedical Sciences Research Center ‘Alexander Fleming’, Vari, Greece; 2 Institute of Applied Biosciences, Centre for Research & Technology Hellas, Thessaloniki, Greece; 3 Department of Nutrition and Dietetics, School of Health Science and Education, Harokopio University, Athens, Greece; 4 Institute for Fundamental Biomedical Science, Biomedical Sciences Research Center ‘Alexander Fleming’, Vari, Greece; 5 Hellenic Naval Academy, Hatzikyriakou Avenue, Pireaus, Greece; 6 Pediatric Hematology/Oncology Unit (POHemU), First Department of Pediatrics, University of Athens, Aghia Sophia Children’s Hospital, Athens, Greece; 7 Wellcome Sanger Institute, Hinxton, UK; 8 Department of Genetic Medicine and Development, University of Geneva Medical School, Geneva, Switzerland; 9 Swiss Institute of Bioinformatics, University of Geneva, Geneva, Switzerland; 10 Institute of Genetics and Genomics in Geneva, University of Geneva, Geneva, Switzerland; 11 Center of New Biotechnologies & Precision Medicine, Medical School, National and Kapodistrian University of Athens, Athens, Greece; 12 First Department of Surgery, National and Kapodistrian University of Athens, Medical School, Laiko Hospital, Athens, Greece; 13 Department of Human Genetics, McGill University Genome Centre, McGill University, Montréal, QC Canada; 14 Department of Bioengineering, McGill University, Montréal, QC Canada

**Keywords:** Adipose tissue, eQTLs, Greek population, Modest-sized studies, Environmental effects

## Abstract

**Background:**

Expression quantitative trait loci (eQTL) studies provide insights into regulatory mechanisms underlying disease risk. Expanding studies of gene regulation to underexplored populations and to medically relevant tissues offers potential to reveal yet unknown regulatory variants and to better understand disease mechanisms. Here, we performed eQTL mapping in subcutaneous (S) and visceral (V) adipose tissue from 106 Greek individuals (Greek Metabolic study, GM) and compared our findings to those from the Genotype-Tissue Expression (GTEx) resource.

**Results:**

We identified 1,930 and 1,515 eGenes in S and V respectively, over 13% of which are not observed in GTEx adipose tissue, and that do not arise due to different ancestry. We report additional context-specific regulatory effects in genes of clinical interest (e.g. oncogene *ST7*) and in genes regulating responses to environmental stimuli (e.g. *MIR21, SNX33*). We suggest that a fraction of the reported differences across populations is due to environmental effects on gene expression, driving context-specific eQTLs, and suggest that environmental effects can determine the penetrance of disease variants thus shaping disease risk. We report that over half of GM eQTLs colocalize with GWAS SNPs and of these colocalizations 41% are not detected in GTEx. We also highlight the clinical relevance of S adipose tissue by revealing that inflammatory processes are upregulated in individuals with obesity, not only in V, but also in S tissue.

**Conclusions:**

By focusing on an understudied population, our results provide further candidate genes for investigation regarding their role in adipose tissue biology and their contribution to disease risk and pathogenesis.

**Supplementary Information:**

The online version contains supplementary material available at 10.1186/s12864-023-09532-w.

## Background

Adipose tissue is a medically relevant tissue and contributes to the pathogenesis of multiple diseases, including type 2 diabetes, cardiovascular disease and cancer [1]. Moreover, it is a tissue of extreme plasticity, responding to environmental cues, such as excess nutrient intake, energy overload, changes in temperature and air pollution [2, 3]. White adipose tissue represents the majority of adipose tissue mass in humans and consists of two main types: subcutaneous (S) and visceral (V) fat. Although S and V fat share a large proportion of their biology, there are important functional differences, including inflammatory and metabolic functions (e.g. lipolysis rate, insulin sensitivity, adipokine secretion) [4, 5]. A fraction of these differences arise from differences in patterns of gene expression [6, 7], an intermediate phenotype that is shaped by genetic, epigenetic and environmental factors. Genetic factors that influence gene expression have been shown to play a key role in shaping complex traits and determining risk for disease [8]. Expression quantitative trait loci (eQTL) studies to date have identified regulatory variants that are active across tissues [9–12], and have highlighted that eQTLs are enriched among disease genome-wide association study (GWAS) signals [9]. Adipose tissue eQTLs, for example, are enriched among cardiometabolic (e.g. body mass index (BMI), waist-to-hip ratio (WHR), circulating lipid levels) [13–16] and neurological (e.g. schizophrenia) [17] traits. Furthermore, studies of epigenetic traits such as chromatin accessibility profiling (e.g. Assay for Transposase-Accessible Chromatin using sequencing (ATAC-Seq)) in adipose tissue have revealed regulatory genomic regions with functional roles in cardiometabolic disease [18]. Thus, linking regulatory variation to GWAS findings contributes to our understanding of disease mechanisms by highlighting specific genes in specific tissues as likely mediators of the disease associations.

The Genotype-Tissue Expression Project (GTEx) is a key resource that has added to our understanding of the action of regulatory variants across human tissues [9]. GTEx participants are mainly of Central and Northern European descent, with ~ 14% of participants being individuals of African-American or Asian-American ancestry, who live in the US. In addition to genetic ancestry [19], gene expression is affected by environmental exposures which can modulate regulatory associations between variants and phenotypes [20–23]. Expanding studies of gene regulation to include underexplored populations and populations living in different environmental conditions therefore is a strategy that is expected to reveal additional, context-specific regulatory variants, building on resources such as GTEx.

In the present study we explored adipose tissue gene expression in Greek individuals living in Central and Southern Greece. We performed eQTL mapping in S and V tissues from 106 individuals and compared findings to GTEx. We report tissue and population differences in the regulatory landscape underlying disease risk and we argue that such differences may arise due to variation in gene expression that is driven in part, by different environmental exposures.

## Results

### GM individuals map close to Italian and Spanish populations

Principal component analysis (PCA) revealed that GM individuals map close to the Tuscan (TSI) and Iberian (IBS) 1000 Genomes (1 KG) populations (Additional File 1: Supplementary Figure S1). This finding is in line with our previous work on coding variants [24] and with results from studies reporting genetic similarity between Greek and Italian subpopulations [25, 26].

### eQTL identification in GM and GTEx

**Fig. 1:**
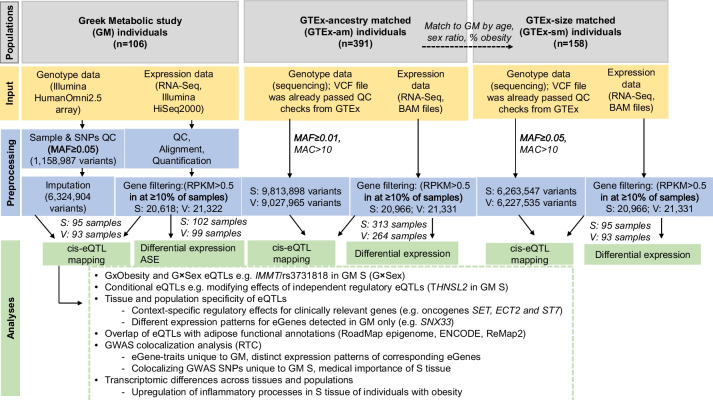
Flowchart outlining the study design and analysis. ASE: allele-specific expression. MAC: minor allele count. MAF: minor allele frequency. eQTL: expression quantitative trait locus. RTC: Regulatory Trait Concordance. RPKM: Reads Per Kilobase Million. S: subcutaneous; V: visceral

In GM, association of ~ 6 M single nucleotide polymorphism (SNP) genotypes (minor allele frequency; MAF ≥ 0.05) with expression levels of 20,618 (S) and 21,322 (V) genes yielded 1,930 and 1,515 eGenes (genes with at least one cis-eQTL) in S and V respectively (1,047 shared) (Table 1, Fig. 1, Additional File 2: Supplementary Table S1). Seven out of the top ten eGenes were identified in both tissues (Table 2).

**Table 1:** Comparison of eQTL mapping results from GM and GTEx-am

**Across tissues (S vs. V)**			
**GM**	**eGenes**	**eQTLs**	**eQTL-eGene pairs**
S	1,930	1,847	1,930
V	1,515	1,448	1,515
overlapping	1,047	237	232
**GTEx-am**	**eGenes**	**eQTLs**	**eQTL-eGene pairs**
S	8,638	8,158	8,638
V	6,151	5,809	6,151
overlapping	4,743	853	854
**Across populations (GM vs. GTEx)**			
**S**	**eGenes**	**eQTLs**	**eQTL-eGene pairs**
GM	1,930	1,847	1,930
GTEx-am	8,638	8,158	8,638
overlapping	1,673	190	178
**V**	**eGenes**	**eQTLs**	**eQTL-eGene pairs**
GM	1,515	1,448	1,515
GTEx-am	6,151	5,809	6,151
overlapping	1,283	133	123

Number of eQTLs detected in each tissue is lower than the number of eQTL-eGene pairs as an eQTL may be associated with more than one gene

*GM* Greek Metabolic study, *GTEx* Genotype-Tissue Expression, *GTEx-am* GTEx-ancestry matched sample, *eQTL* expression quantitative trait locus, *eGene* gene with at least one cis-eQTL

**Table 2:** Top 10 cis-eQTLs in GM S and V tissues

**GM S**							
**eQTL**	**Chr**	**MA**	**MAF**	***P*-value**	**Q-value**	**GeneID**	**Gene name**
rs3965185	19	T	0.489474	3.87E-36	5.00E-32	ENSG00000233927.4	*RPS28*
rs7308368	12	T	0.415789	2.98E-30	1.93E-26	ENSG00000013573.12	*DDX11*
rs59212603	7	C	0.236842	9.30E-27	4.00E-23	ENSG00000226278.1	*PSPHP1*
rs10096524	8	G	0.415789	2.16E-26	6.96E-23	ENSG00000071894.10	*CPSF1*
rs2687969	4	T	0.321053	9.99E-26	2.58E-22	ENSG00000163682.11	*RPL9*
rs12366	2	T	0.484211	7.02E-25	1.51E-21	ENSG00000204792.2	*AC104135.3*
rs1729660	2	T	0.363158	1.18E-24	2.17E-21	ENSG00000237651.2	*C2orf74*
rs393329	16	T	0.4	7.55E-24	1.22E-20	ENSG00000234719.4	*RP11-166B2.1*
rs2927608	5	A	0.326316	1.36E-23	1.95E-20	ENSG00000164308.12	*ERAP2*
rs35725606	6	T	0.478947	2.32E-23	3.00E-20	ENSG00000203875.6	*SNHG5*
**GM V**							
**eQTL**	**Chr**	**MA**	**MAF**	***P*-value**	**Q-value**	**GeneID**	**Gene name**
rs2687969	4	T	0.322581	5.21E-30	7.35E-26	ENSG00000163682.11	*RPL9*
rs1046456	12	T	0.413978	2.76E-29	1.95E-25	ENSG00000013573.12	*DDX11*
rs3965185	19	T	0.494624	1.40E-28	6.59E-25	ENSG00000233927.4	*RPS28*
rs2927608	5	A	0.327957	1.33E-25	4.60E-22	ENSG00000164308.12	*ERAP2*
rs9928222	16	C	0.456989	1.63E-25	4.60E-22	ENSG00000059122.12	*FLYWCH1*
rs12366	2	T	0.483871	5.20E-25	1.22E-21	ENSG00000204792.2	*AC104135.3*
rs11021552	11	T	0.241935	1.21E-24	2.44E-21	ENSG00000149231.7	*CCDC82*
rs59212603	7	C	0.236559	3.39E-24	5.97E-21	ENSG00000226278.1	*PSPHP1*
rs10406056	19	C	0.354839	1.08E-23	1.69E-20	ENSG00000174652.13	*ZNF266*
rs1729660	2	T	0.365591	1.57E-23	2.21E-20	ENSG00000237651.2	*C2orf74*

All cis-eQTL results are provided in S Table 1

*Chr* chromosome, *MA* minor allele, *MAF* minor allele frequency, *P-value* significance in beta permutation (fastQTL), *Q-value* false discovery rate, *GM* Greek Metabolic, *S* subcutaneous, *V* visceral

Over 70% of eGenes detected are protein-coding, eQTLs cluster around the transcription start site (TSS), and show consistent allelic direction across tissues (Additional File 1: Supplementary Figure S2A-C). Despite modest sample size, conditional analysis in GM revealed 76 and 45 eGenes with a secondary, independent eQTL in S and V respectively. Secondary eQTLs tend to be located more distal to the TSSs of associated genes compared to primary eQTLs (Additional File 1: Supplementary Figure S2D).

In GTEx-am, association of ~ 9 M SNP genotypes with 20,966 (S) and 21,331 (V) genes yielded 8,638 and 6,151 eGenes in S and V respectively (4,743 shared) (Table 1). Direction of allelic effects was consistent across GM and GTEx-am for each tissue (Additional File 1: Supplementary Figure S3). Similar numbers of eGenes were also detected for the GTEx-size-matched (GTEx-sm) sample (Supplementary Material 1: Supplementary Table S2, Supplementary Text S1).

To identify regulatory effects linked to obesity and to sex, we performed eQTL mapping in GM, using a linear model with an interaction term (Genotype × Obesity and Genotype × Sex). We detected 117 and 77 Genotype × Obesity (Additional File 2: Supplementary Table S3) and 114 and 92 Genotype × Sex (Additional File 2: Supplementary Table S4) regulatory interactions in S and V respectively, with nominal *P* < 0.05, but none survived false discovery rate (FDR) < 5%. In GM S for example, genotypes at rs3731818, an eQTL for *IMMT* (Inner Membrane Mitochondrial Protein), showed interaction with sex (P_Genotype×Sex_ = 8.58e-04) (Additional File 1: Supplementary Figure S4). Rs3731818 is also associated with systolic blood pressure [27]. In adipose tissue, mitochondrial dysfunction has been linked to inflammation and metabolic disease, including hypertension [28]. This potential, subtle effect of sex on disease risk through *IMMT* expression regulation comprises an angle worth investigating in future studies. When obesity status was included as a covariate in our eQTL analysis, we replicated ~ 97% of our discoveries (Additional File 2: Supplementary Table S5).

To further explore *cis* effects on gene regulation in GM, we tested for allele-specific expression (ASE). We detected 110 and 115 ASE SNPs (77 shared) mapping in 112 and 118 genes (87 shared) in S and V respectively. Functional characterisation of ASE sites revealed ten missense, likely pathogenic SNPs in each tissue (five shared) (Additional File 2: Supplementary Table S6), including SNPs in *FMO2* (Flavin Containing Dimethylaniline Monoxygenase 2) and *SULT1A2* (Sulfotransferase Family 1A Member 2), genes involved in drug and xenobiotic metabolism. *FMO2* belongs to a family of xenobiotic-metabolizing enzymes that improve resistance to a broad range of environmental stressors, such as chemicals and drugs [29]. The *FMO2* ASE SNPs rs2020862 and rs2020870 encode deleterious amino acid substitutions (S195L and D36G respectively) suggesting that distinct FMO2 isoforms exist at different levels in S. Disentangling the mechanics of gene regulation in similar cases will aid our understanding of complex phenomena such as disease penetrance where the effect of a coding variant may be modified by co-existing regulatory effects.

### Properties and functional characterisation of eQTLs

**Fig. 2:**
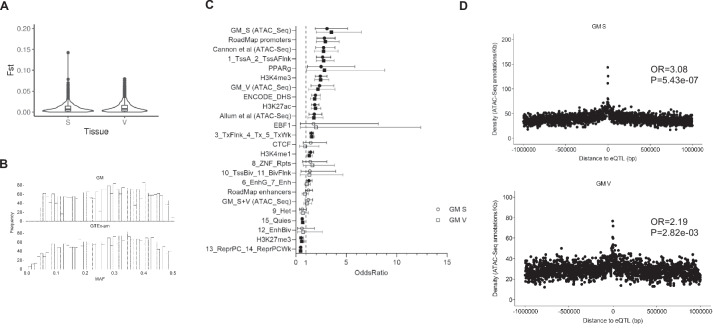
Properties of GM detected eQTLs. **A** Violin plot of Fst values for GM detected eQTLs in each tissue compared to GTEx-am. **B** MAF histogram plots showing allele frequencies of eQTLs in GM and GTEx-am. Among 3,058 GM-detected eQTLs in both tissues, 2,939 were also available in GTEx-am dataset (of which, 2,610 had the same minor allele). MAF comparison across populations revealed no differences (*P* = 0.33; M-W test). **C** Enrichment of GM eQTLs in adipose tissue functional annotations is shown as estimated odds ratios and 95% confidence intervals on the x axis for each annotation category in the y axis. Odds ratios greater than 1 indicate an enrichment of eQTLs in the given functional annotations, while odds ratios less than 1 indicate a depletion. Significant odds ratios are shown as filled circles or squares (*P* < 0.05). Cannon et al. (ref 18), Allum et al. (ref 30). **D** Enrichment of GM eQTLs in GM ATAC-Seq peaks in S (upper panel) and V (bottom panel) tissue

To test whether differences in eQTLs across populations arise from differences in allele frequencies, we calculated fixation index (Fst) for all GM-detected eQTLs. We detected no prominent differentiation in allele frequencies (mean Fst in S and V ~ 0.0044) (Fig. 2A), suggesting that eQTLs detected only in GM are not driven by allele frequency differences between GM and GTEx. Indeed, no differences in MAF distribution of eQTLs across GM and GTEx-am were found (*P* = 0.33; M-W test, Fig. 2B).

To characterise the functional impact of GM eQTLs, we tested their overlap with functional annotations in adipose tissue from RoadMap Epigenomics, ENCODE, Remap2 and from ATAC-Seq publications [18, 30]. We found that eQTLs were significantly enriched in transcriptionally active histone marks (H3K4me3, *P* = 8.65e-15 in S and *P* = 1.86e-11 in V and H3K27ac, *P* = 1.38e-10 in S and *P* = 9.01e-09 in V, Fisher’s exact test) and significantly depleted from repressive histone marks (H3K27me3, *P* = 3.07e-04 in S and *P* = 6.18e-03, Fisher’s exact test) (Fig. 2C). GM eQTLs were also enriched in GM ATAC-Seq peaks for S (*P* = 5.43e-07, Fisher’s exact test) and V (*P* = 2.82e-03, Fisher’s exact test) tissue (Fig. 2D). Similarly to GM, GTEx-am eQTLs were enriched in functional annotations (Additional File 1: Supplementary Figure S5).

### Genetic regulatory effects across tissues and populations: eQTL sharing and specificity

#### Across tissues

Given that the FDR-based comparisons shown in Table 1 likely underestimate the extent of shared effects (54–69% overlap with 1,047 shared eGenes across tissues), we used p-value enrichment analysis (pi1 estimate for replication, [31]) to obtain a better understanding of sharing of eQTLs between S and V. We report a replication rate of ~ 93%, indicating highly shared mechanisms of gene regulation across tissues, in accordance with previous studies [32]. However, we detected 83 eQTL-eGene pairs only in S and 62 only in V, through lack of association in the replicating tissue (based on the 5% tail of association p-values) (Additional File 2: Supplementary Table S7). We extended our investigation of tissue specific associations through a linear model incorporating a Genotype × Tissue interaction term, and we report 200 eQTL-eGene pairs (177 eGenes) with a significant interaction term (FDR < 5%) (Additional File 2: Supplementary Table S8). Thirty-five eGenes (same number of eQTLs), were identified by both methods (Additional File 2: Supplementary Table S9) further supporting tissue specific regulatory effects. Twenty-one of these eGenes were detected only in GM and include genes with a role in diseases such as cancer (e.g. *LUZP6* [33] *PCDHB13* [34]) and obesity (e.g. *NTRK2* [35]), narrowing down the list of genes that constitute promising candidates for further study. Four of the above eQTLs were also detected through our GxObesity analysis (rs62015152-*CLN6*, rs3805695-*PCDHB13*, rs12378391-*CCDC183* and rs2516064-*SUCO*). Notably rs12378391, an eQTL in GM S, colocalizes with GWAS SNP rs12380852, that is linked to waist circumference (adjusted for BMI).

#### Across populations

**Fig. 3:**
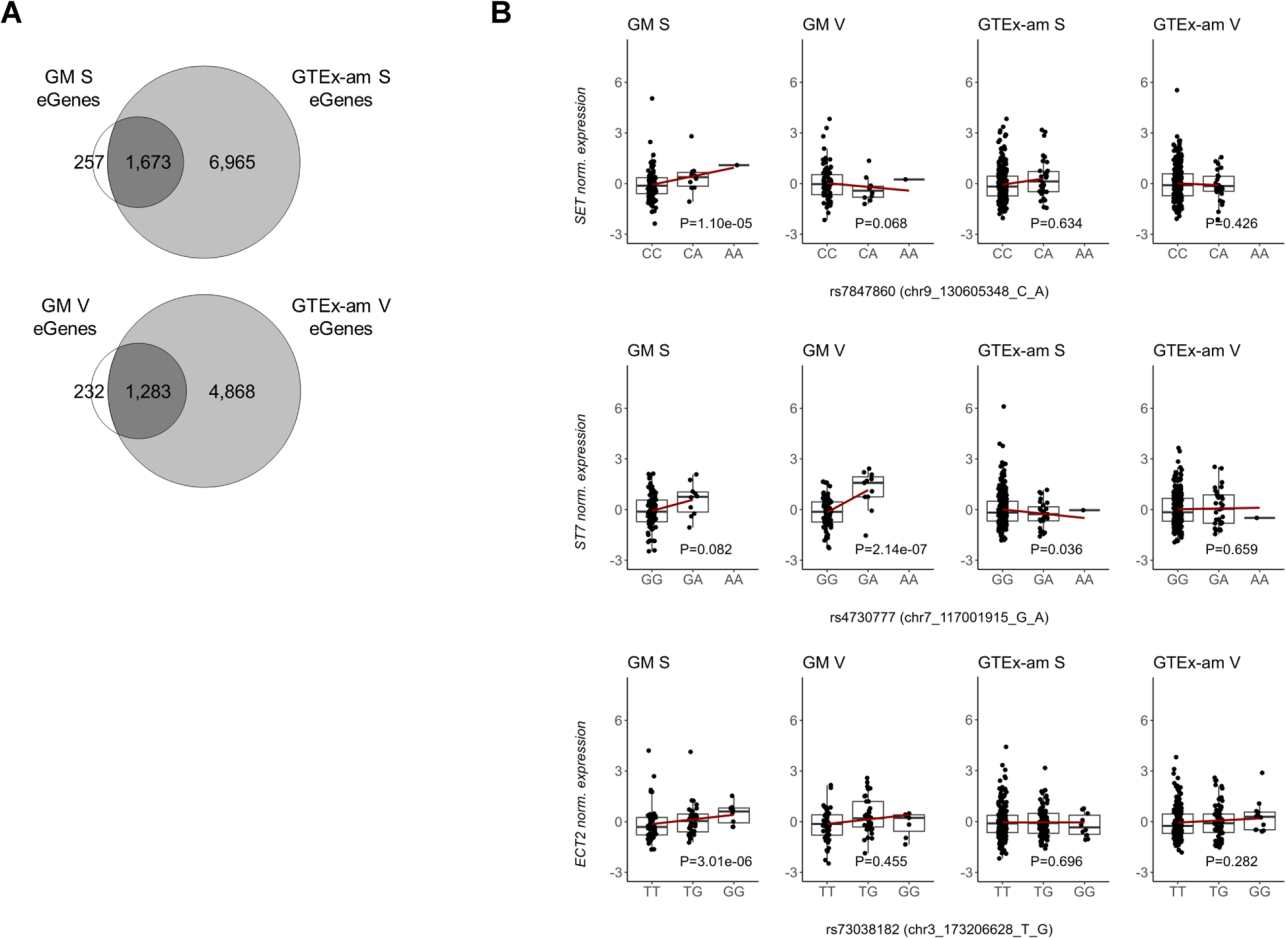
Population sharing of GM detected eQTLs. **A** Overlap of eGenes detected in GM and GTEx-am in S and V. **B** eQTL plots for the oncogenes *SET, ECT2 and ST7*, showing population-specific regulatory effects. These eQTLs were found in GM only based on pi1 analysis. *P*-values represent association nominal p-values. GM: Greek Metabolic; GTEx-am: GTEx-ancestry-matched; S: subcutaneous; V: visceral

We applied the same approach to compare findings across populations. FDR-based comparisons of eGenes shown in Table 1 (GM vs GTEx-am) revealed an overlap of 85–87%, with 1,673 and 1,283 shared eGenes for S and V tissues respectively (Fig. 3A). Through pi1 analysis, we detected replication rates of GM associations in GTEx-am ranging from 90% (V) to 96% (S), implying common mechanisms of gene regulation, in line with similar studies [16]. Despite high levels of replication, we detected 78 (S) and 61 (V) eQTL-eGene pairs in GM only (139 eGenes in total, of which 64 were not identified as eGenes in GTEx-am), given the lack of association in GTEx-am (based on the 5% tail of association p-values) (Additional File 2: Supplementary Table S10). Given low Fst values (mean Fst across populations for S and V ~ 0.0039), we argue that eQTLs detected in GM only, do not arise from differences in allele frequencies across populations. Although we did not detect enrichment of biological properties (through gene ontology (GO) terms) amongst associated eGenes, we report clinically relevant genes including oncogenes *SET, ST7* and *ECT2* in GM only (Fig. 3B).

To further explore specificity at the population level, we focused on GM-detected eGenes. Of the 1,047 eGenes detected in both GM tissues, 32 were not identified in GTEx-am (Additional File 2: Supplementary Table S11), with corresponding eQTLs not displaying allele frequency differences (mean Fst in S and V ~ 0.0046). Of these eGenes seven display very different patterns of gene expression across populations (but similar in both tissues for each population) that may arise in part due to environmental factors (Additional File 1: Supplementary Figures S6, S7, S8). GM eGene *SNX33* for example encodes a protein regulating cellular responses to environmental stimuli (including nutrient uptake), developmental regulation and cell signaling [36, 37]. Given that sorting nexin (SNX) family proteins have a role in the pathophysiology of diseases such as cardiovascular and neurodegenerative disease [38, 39] we suggest that follow-up studies focusing on the regulation of this gene may help elucidate its potential involvement in disease risk.

#### Across tissues and populations

**Fig. 4:**
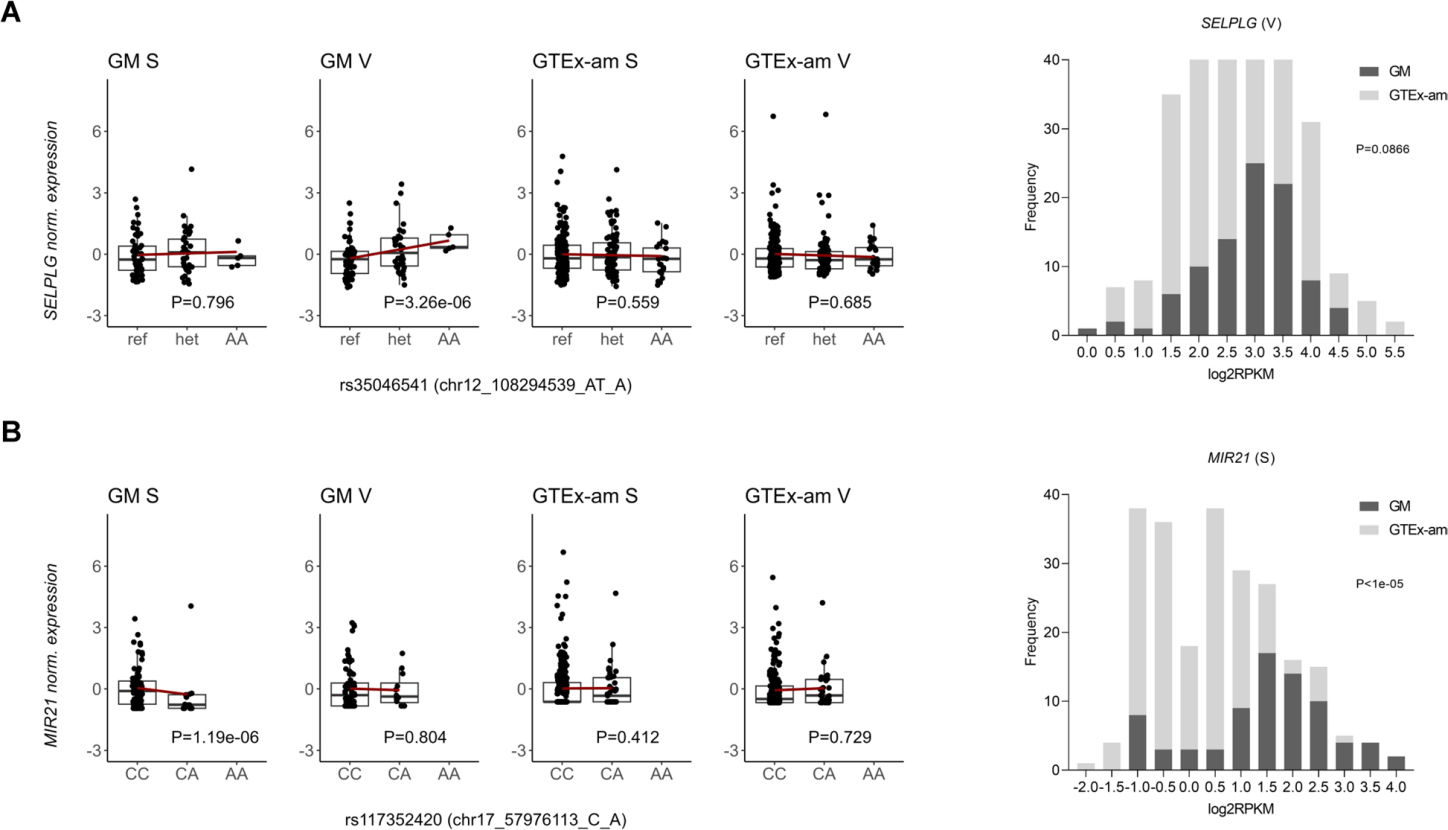
Examples of GM detected eQTLs showing tissue and population specific regulatory effects. **A** *SELPLG*/rs35046541 eQTL, example of an eQTL detected in a single tissue (V) in GM only, that colocalizes with rs1895941, a GWAS SNP for BMI. Expression levels of SELPLG across GM and GTEx-am in V tissue are shown in the histogram (P-value from M-W test). **B** *MIR21*/rs117352420 eQTL, examples of an eQTL detected in GM S only and where expression patterns of the eGene differ across GM and GTEx-am (histogram, P-value from M-W test). GM: Greek Metabolic; GTEx-am: GTEx-ancestry-matched; S: subcutaneous; V: visceral

Finally, we sought to identify eQTL-eGene pairs that were detected in a single tissue in GM only, and report 23 and 20 such associations in GM S and V respectively. We investigated colocalization of these eQTLs with GWAS SNPs and report four instances of overlap in each tissue. We highlight rs35046541, an eQTL for *SELPLG* (Selectin P Ligand) detected in GM V only, that colocalizes with rs1895941, a SNP associated with BMI [40] (Fig. 4A). *SELPLG* is a gene with a role in immune cell trafficking during inflammation [41] and has been associated with adiposity and obesity [42]. For the above eQTL, no allele frequency differentiation between GM and GTEx-am was detected (Fst = -0.0027), while *SELPLG* displayed distinct expression patterns in V tissues (GM V, mean RPKM = 8.67; GTEx-am V, mean RPKM = 8.34, *P* = 0.086, M-W test). We suggest that expression differences may be linked to environmental factors that drive the action of context-specific regulatory variants.

Similarly, we report *MIR21* (MicroRNA 21) and associated eQTL rs117352420 detected in GM S only (Fig. 4B**, **Additional File 2: Supplementary Tables S7, S10). *MIR21* displays distinct expression patterns in GM S vs. GTEx-am S (GM S mean RPKM = 3.23; GTEx-am S mean RPKM = 0.69, *P* < 1e-05, M-W test, Fig. 4B) and is one of most frequently upregulated miRNAs in solid tumors [43]. We report that eQTL rs117352420 colocalizes with GWAS SNPs linked to pulse pressure, haematological traits and immune-related diseases including inflammatory bowel disease (IBD) and multiple sclerosis (Additional File 2: Supplementary Table 12).

### GWAS-eQTL colocalizations: similarities and differences across populations

**Fig. 5:**
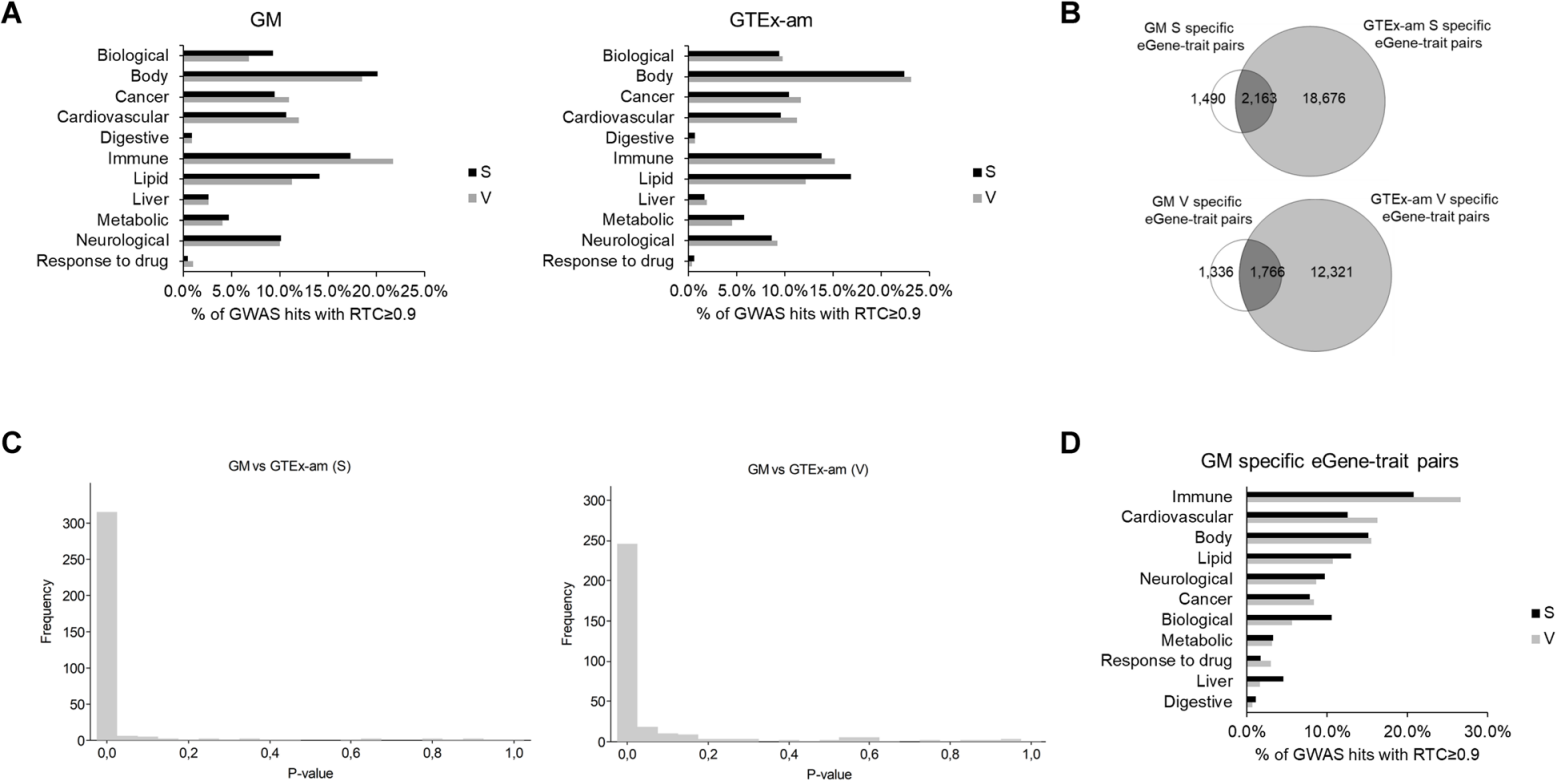
Colocalization of eQTLs with GWAS signals in GM and GTEx. **A** EFO bar charts for eQTLs colocalizing with GWAS signals in GM and GTEx-am. **B** Population overlap of colocalized eGene-trait pairs, per tissue. **C** Histogram of M-W p-values from comparison of expression levels of 364 (of which 16 were not expressed in GTEx-am S) and 343 (of which 18 were not expressed in GTEx-am V) eGenes corresponding to GM specific eGene-trait pairs. **D** Percentages of EFO categories for eGene-trait pairs detected only in GM. GM: Greek Metabolic; GTEx-am: GTEx-ancestry-matched; S: subcutaneous; V: visceral

To address how adipose tissue regulatory variation contributes to complex traits and disease, we explored colocalization of GM-detected eQTLs with 99,655 GWAS SNPs. We found that approximately half of detected eQTLs (47.3% in S and 50.5% in V) colocalize with GWAS SNPs, highlighting potential causal genes and their tissue of action (Table 3, Additional File 2: Supplementary Table S12). Despite sample size differences, similar levels of GWAS-eQTL colocalization were observed for GTEx-am (~ 54%) (Table 3). Of note, over 35% of eQTLs colocalize with GWAS SNPs linked to 117 cardiometabolic traits (Supplementary Material 1: Supplementary Table S13, Supplementary Text S3). Furthermore, following grouping of GWAS associated traits by experimental factor ontology (EFO) category, we found “body measurement” and “immune system disorder” as top terms for both GM tissues, followed by “neurological”, “metabolic” and “cancer” associations (Fig. 5A). The prominence of these EFO categories likely reflects the presence of different cell types in adipose tissue and their differential contribution to complex phenotypes [44, 45]. Our findings are in line with studies from larger datasets (e.g. METSIM [16]) and highlight the use of modest-sized studies in understudied populations to help build on existing knowledge.

**Table 3:** Number of eQTLs colocalizing with GWAS SNPs in GM and GTEX-am

Population sample	Input eQTLs	Input genes	Input GWAS-SNPs	Tested eQTLs	Colocalized eQTLs^a^	Colocalized GWAS-SNPs	% of colocalized eQTLs^b^
GM S	1,847	20,618	99,655	1,371	648	2,290	47.30%
GM V	1,448	21,322	99,655	1,092	552	1,955	50.50%
GTEx-am S	8,158	21,692	99,655	6,175	3,374	10,530	54.60%
GTEx-am V	5,809	22,058	99,655	4,404	2,313	7,706	52.50%

*GM* Greek Metabolic, *GTEx-am* GTEx-ancestry-matched, *S* subcutaneous, *V* visceral

^a^Regulatory trait concordance (RTC) ≥ 0.9 for colocalization

^b^Refers to the % of tested eQTLs colocalizing with GWAS SNPs

To uncover population differences in GWAS-eQTL colocalizations, we initially focused on eGene-trait pairs. Over half (57%) of eGene-traits were shared across populations in both S and V. However, we report that 1,490 (41%) and 1,338 (43%) eGene-trait pairs in GM S and V respectively were not replicated in GTEx-am, suggesting population differences in gene regulatory mechanisms underlying complex traits (Fig. 5B**, **Additional File 2: Supplementary Table S14). For these eGene-trait pairs detected in GM only, we explored expression patterns of the corresponding eGenes (364 in S and 343 in V) across populations and we found that over 75% of genes exhibit significantly different expression patterns (Fig. 5C, Additional File 2: Supplementary Table S15). These differences may reflect environmental effects that drive context-specific regulation of underlying traits (493 associated in S and 243 in V) that map to EFO categories including “immune system disorder” (~ 24%), “body measurement” (~ 15%) and “cardiovascular” (~ 14%) (Fig. 5D). We also explored if colocalizing GWAS SNPs differ across populations and found that over 64% of GWAS SNPs were shared across GM and GTEx-am in both tissues (Additional File 1: Supplementary Figure S9A). GWAS associated traits map broadly to the same EFO categories in both tissues. However, for GM S specific GWAS SNPs only, we report an enrichment of ‘cardiovascular’ (*P* = 1.07e-02, OR = 1.65) or depletion of ‘cancer’ (*P* = 8.60e-03, OR = 0.59) and ‘metabolic’ (*P* = 2.47e-02, OR = 0.53) EFO categories (Additional File 1: Supplementary Figure S9B), highlighting the medical importance of S tissue. We hypothesize that GM specific GWAS SNPs colocalizations may partly reflect exposures that are particular to the living environment of the GM population sample. This is important as modulation of these associations through distinct expression patterns contributes to the effect of environmental factors to clinical traits [20].

### GM study provides further knowledge on the fine-tuning of adipose gene regulation

For eGenes with secondary, independent eQTLs (76 in S and 45 in V), we evaluated colocalization of the secondary eQTL with GWAS SNPs. We report 13 and 10 eQTL colocalizations in S and V respectively, with the secondary, but not the primary eQTL, implying a role for regulatory fine-tuning (Additional File 2: Supplementary Table S16). In GM S for example, rs4246598, a GWAS SNP associated with C-reactive protein (CRP) levels [46], colocalizes with the secondary eQTL detected for *THNSL2* (Threonine Synthase Like 2) (rs34185550; RTC = 0.919 and linkage disequilibrium (LD) D΄ = 0.911) (Additional File 1: Supplementary Figure S10). This gene encodes a threonine synthase-like protein that exacerbates inflammation during inflammatory conditions [47] and has been associated with obesity and fat mass [48]. These findings suggest that modifying effects of independent regulatory effects can contribute to differential penetrance of disease variants. Of note, secondary eQTLs colocalize with GWAS SNPs, over half of which (57%) did not colocalize with any primary eQTLs (Additional File 2: Supplementary Table S16). This suggests that secondary eQTLs capture additional biological pathways.

### Transcriptomic and epigenetic analyses reveal known pathways of adipose biology and highlight the medical importance of S tissue

We compared expression levels across S and V and report 2,979 and 5,320 differentially expressed genes (DEGs) in GM and GTEx-am respectively (Additional File 3: Supplementary Table S17). DEGs were involved chiefly in developmental, signalling and inflammatory/immune system-related processes (Additional File 4: Supplementary Figures S11A-B). DEGs by tissue overlapped significantly across populations (of the 2,979 GM-detected DEGs, 1,654 were also detected in GTEx-am, *P* = 1e-288, Fisher’s exact test), suggesting that a substantial fraction of gene expression differences across the two tissues can be captured through studies like GM. Amongst overlapping DEGs, we report 151 instances (Additional File 3: Supplementary Table S18) of discordant direction of gene expression levels. Notably, these genes were mostly involved in biological processes including oxygen transport (e.g. hemoglobin genes *HBB* and *HBG2*) and other responses to environmental stimuli, such as nutrient-sensing pathways (e.g. low-density lipoprotein receptor *LDLR*), suggesting that discordant effects may be at least in part due to environmental differences (Additional File 4: Supplementary Figure S12). In GM, and within each tissue, we also interrogated expression differences by obesity status and sex. When comparing by obesity status, we detected 1,054 and 429 DEGs in S and V respectively (Additional File 3: Supplementary Table S19). For individuals with obesity, we detected upregulation of genes involved in inflammatory processes, not only in V, but in S as well (Additional File 4: Supplementary Figure S13), highlighting the underappreciated clinical relevance of S adipose tissue. When investigating expression differences by sex we found 131 and 40 DEGs in S and V respectively, but no significant enrichment of functions.

Comparison of gene expression levels across populations for each tissue revealed four times as many DEGs (Additional File 3: Supplementary Table S20) highlighting genes involved in developmental, inflammatory, and metabolic processes (Additional File 4: Supplementary Figures S11C-D).

To further characterise adipose tissue in GM, we profiled chromatin accessibility through ATAC-Seq and identified 16,907 and 14,700 peaks of open chromatin in S and V respectively (7,123 shared) (Additional File 3: Supplementary Table S21). In both tissues, genes assigned to peaks were mostly involved in metabolic processes (Additional File 4: Supplementary Figures S14A-B). To detect more general adipose tissue signatures, we pooled reads from all samples (S and V) and detected 121,673 peaks (Additional File 3: Supplementary Table S21). Genes assigned to peaks were mostly involved in developmental and signalling processes (Additional File 4: Supplementary Figure S14C), similar to signatures revealed when comparing gene expression patterns across tissues.

## Discussion

The genetic architecture of gene regulation differs across tissues and populations [10, 12, 19, 20]. These differences contribute to phenotypic variance in complex traits, and in disease risk and pathogenesis. In the present study, we performed eQTL mapping in S and V adipose tissue, in a Greek population sample and compared findings to those from GTEx.

Through the study of an underexplored population, we have uncovered context-specific regulatory variants in genes that shape complex traits and influence disease risk. Comparison of regulatory variants across populations revealed regulatory effects in GM only for genes implicated in cancer, including *ST7* [49], *SET* [50] and *ECT2* [51]. *ST7*, for example, is an eGene in GM V only. This gene has an important role in the development of a range of cancer types, including breast and prostate cancer. *ST7*-eQTL rs4730777 colocalizes with esophageal adenocarcinoma GWAS SNP (rs2188554), suggesting that regulation of *ST7* in adipose tissue may be involved in other cancer types. We also highlight context-specific regulatory effects for genes involved in metabolic (e.g. obesity, *NTRK2*, [35]) and neurological (e.g. schizophrenia, *MAN2A1*, [52]) diseases, and for genes with a role in biological processes such as adipogenesis (e.g. *DEPTOR,* [53]). Overall, our findings highlight regulatory effects on genes with key roles for human health, and add to a growing literature indicating the relatively high yield that can be provided by modest-sized studies on understudied populations [10, 12, 19].

Notably, we demonstrate that our findings do not arise primarily from allele frequency differences across the two populations. Rather, we hypothesize that a fraction of regulatory variants detected arises through environmental effects on gene expression. For example, expression patterns for *MIR21*, the most commonly upregulated microRNA in solid tumors, differ significantly between GM and GTEx. We report an eQTL association in GM S only. MicroRNAs have been increasingly used in studying environmental exposures and health effects [54] and are commonly deregulated by various types of environmental pollutants, including airborne contaminants [55]. MIR21 is thought to act on small blood vessels, mediating the effects of air pollution that lead to endothelial dysfunction and to cardiac disease [56]. Studies in mice and humans have demonstrated its involvement in the development of heart disease [57], with higher MIR21 levels detected in murine and human hearts [58]. *MIR21* is negatively affected by exposure to certain air pollutants (e.g. particulate matter PM2.5 and PM10, black carbon, organic carbon, and sulphates), as shown in human and animal-based studies [55, 56, 59]. Given this inverse association of *MIR21* expression with exposure to air pollutants, the higher MIR21 expression observed in GM S may arise due to exposure to lower levels or different types of airborne particulate matter compared to GTEx individuals. We report that the eQTL identified for *MIR21* colocalises with GWAS SNPs for pulse pressure, IBD, and multiple sclerosis (Additional File 2: Supplementary Table S12) traits that are influenced in part by air pollution [60–62]. Air pollution (airborne particulate matter) exerts negative effects on the human skin [63, 64] and has been linked with inflammation in subcutaneous adipose tissue [65]. In addition to respiratory uptake of air pollutants, recent work suggests that there may be direct effects of particulate matter on S adipose tissue with air pollution particles reaching this tissue through hair follicles in the human skin [64]. Such connections between gene regulation, environmental effects and disease risk are a first step towards unravelling mechanisms of pathogenesis and the accompanying contribution of environmental factors. Furthermore, such examples demonstrate the value of studying populations living in different environmental conditions. Such likely contributing factors are differences in air pollution levels (e.g. *MIR21*, [20]) or in dietary intake (e.g. *LDLR*, key regulator of cholesterol uptake, which showed discordant direction of gene expression levels between GM and GTEx-am, Additional File 3: Table S18).

We also report seven eGenes identified only in GM which display significant expression pattern differences across populations. *SNX33*, for example, belongs to the family of sorting nexins (SNXs), that modulate responses to environmental stimuli such as nutrient uptake, by shaping the sub-cellular localization of different nutrient receptors [39]. SNXs have been implicated in neurological diseases including Alzheimer’s disease (AD) [39]. Recently SNX33 was found to be upregulated by the anti-AD drug donepezil, positioning this gene as a promising therapeutic target for the disease [66]. In GM we report overall lower *SNX33* expression levels in both tissues compared to GTEx. Understanding how environmental influences shape expression patterns of such drug target genes is critical, and studies such as the present one contribute towards this direction.

We also report upregulation of inflammatory processes (e.g. cytokine production, phagocytosis) in S adipose tissue of individuals with obesity (Additional File 4: Supplementary Figure S13). Furthermore, we demonstrate colocalization of eQTLs detected in GM S with previously reported traits (e.g. BMI, WHR), but also with disease traits that according to our knowledge have not been reported as colocalizing to date, including stroke and Alzheimer disease (Additional File 2: Supplementary Table S12). Recent study in obese mice receiving S tissue lipectomy reported neuroprotective effects of S tissue against brain inflammation, a feature of dementia and stroke [67], highlighting the important role of S tissue in disease pathogenesis. Understanding further the mechanisms of involvement of S tissue in disease risk is of interest and our findings contribute towards this direction.

Although modest sample size is a limitation of the present study, we demonstrate that studies of this size can reveal previously undetected regulatory variants. We report a replication rate of > 90% of our findings in the larger GTEx sample, but also record additional context-specific regulatory effects. Similar studies have shown that population differences in eQTLs stem from differences in allele frequencies [10, 12], while our study suggests that eQTL differences can also arise from environmental effects on gene expression. A second limitation of our study is that gene expression is from bulk adipose tissue, reflecting the overall biology of S and V adipose tissue. Given this, we cannot account for cell-type heterogeneity and its importance as a confounder in the interpretation of disease loci [45]. Cellular heterogeneity is reflected by the prominence of disease categories including immune, metabolic, neurological and developmental signals for detected DEGs, chromatin accessibility genomic regions and GWAS-eQTL colocalization instances. Finally, our study has interrogated genetic variation through genotyping followed by imputation. As a result we have tested fewer variants than GTEx, which includes genetic variation data from DNA sequencing. Therefore, we have most likely missed an important fraction of causal variants [68] compared to GTEx. However, we were able to detect similar levels to GTEx of eQTL enrichment around, and in functional annotations.

## Conclusions

By focusing on an understudied population, we have uncovered adipose tissue regulatory variants that likely arise due to differences in gene expression patterns across GM and GTEx and involve context-specific regulatory effects for clinically relevant genes. Uncovering these eQTLs highlights the utility of modest-sized studies in adding to our understanding of the molecular underpinnings of complex traits and to the identification of mechanisms that drive disease in specific tissues.

## Methods

### Population samples

#### Greek Metabolic (GM) study

GM comprises 106 Greek individuals (54 females), aged 18–85 years (mean age at 53.8yrs) with a BMI range 18–64 kg/m^2^ (47% with obesity, defined as BMI ≥ 30 kg/m^2^) (Supplementary Material 1: Supplementary Table S22). GM participants were individuals who were admitted to Laiko General Hospital in Athens for abdominal surgery (e.g. cholecystectomy, weight reduction surgery). Among 50 individuals with obesity, thirty underwent bariatric surgery. Following informed consent, paired samples of abdominal subcutaneous (S) and visceral (V) adipose tissue were collected. Samples were stored immediately in Allprotect Tissue Reagent (Qiagen, Hilden, Germany) and transferred to -80 °C. The project was approved by the Bioethics Committee of Harokopio University of Athens (38073/13–07/2012), based on the Helsinki Declaration.

#### GTEx samples

We downloaded GTEx (v7) genotype and S and V expression data (478 individuals). To match GM and GTEx for ancestry, we used PCA on GTEx genotypes and retained 391 individuals of European ancestry (GTEx-ancestry-matched sample; GTEx-am) (Supplementary Material 1: Supplementary Table S22). To match the sample size and phenotypic characteristics (age, sex ratio, % of individuals with obesity), we randomly sampled 158 GTEx-am individuals, defining the GTEx-size-matched sample (GTEx-sm) (Fig. 1, Supplementary Material 1: Supplementary Text S1, Supplementary Table S22).

### Genotypes, gene expression, open chromatin

#### GM data

##### Genotyping and imputation

Genomic DNA was extracted from blood using the iPrep PureLink gDNA Blood kit and iPrep Purification Instrument (Invitrogen, Life Technologies, Carlsbad, California, USA). Extracted DNA was genotyped on the Illumina HumanOmni2.5 array (Exome 8v1-A or 8v1-1_b). Sex check by PLINK [69] was performed to identify individuals with discordant sex information. Duplicated samples, related individuals and subjects that did not cluster with 1 KG European populations through PCA were removed. A total of seven individuals were excluded, leaving 99 individuals. Genotypes were pre-phased with SHAPEIT [70] and imputed to the 1 KG Genomes Project Phase III reference panel [71] using IMPUTE 2 [72]. Following imputation, single nucleotide polymorphisms (SNPs) were filtered for minor allele frequency MAF ≥ 0.05, imputation confidence score INFO of > 0.4 and Hardy–Weinberg Equilibrium (HWE) p > 1e-06, yielding ~ 6.3 million variants. PCA on genotypes was carried out using PLINK [69] to determine the extent of GM population structure and to compare GM to other European populations, including 1KG_EUR and the GTEx population samples described above.

##### RNA-Seq

RNA was extracted from S and V samples using the RNeasy Lipid Tissue MiniKit (Qiagen, Hilden, Germany) and libraries were prepared with the Illumina TruSeq kit. Sequencing was performed on the Illumina HiSeq2000 platform at two centers (University of Geneva, paired-end 49 bp reads, 81 samples and Genome Quebec, paired-end 100 bp reads, 129 samples) to a median depth of 53.1 million reads (interquartile range 33–57 million reads). In order to detect known and hidden confounders affecting gene expression, we performed linear mixed model regressions of available technical (e.g. GC content, insert size, sequencing center, RNA integrity number (RIN)) and biochemical (e.g. triglycerides, fasting glucose) variables on gene expression using the lme4 R package [73]. We used the pi1 statistic [31] to detect covariates affecting a large number of genes. We selected the age, sex and BMI category (individuals with or without obesity; BMI >  = 30 kg/m^2^) as the most informative covariates to include in our differential expression analyses. To ensure data comparability, 100 bp reads were trimmed to 49 bp and mapped to GRCh37 using GEM [74]. Libraries with depth < 25 million reads were retained following diagnostic tests to ensure that the available RNA-Seq reads were adequate to detect genes in a homogeneous manner similarly to samples with more reads. To this end we used metaseqR [75] to assess the adequacy of libraries to detect genes (features and biotypes) by constructing sequential curves depicting the percentage of biological features detected when subsampling the total number of reads. Libraries with depth < 25 million reads did not differ in this manner from the rest of the samples and were thus retained. Importantly, Pearson correlation analysis between fold changes across two conditions (including or excluding samples with < 25 million reads) did not reveal substantial differences (Pearson’s R = 0.994). Gene-level quantification was performed on GENCODEv19 using QTLtools *quan* module [76]. We excluded outliers using PCA plot on RPKM (Reads Per Kilobase Million) values, leaving 102 samples in S and 99 in V. PCA revealed no batch effects (Supplementary Material 1: Supplementary Figure S15). Genes with RPKM ≥ 0.5 in at least 10% of individuals were taken forward to further analyses.

##### Open chromatin profiling (ATAC-Seq)

Chromatin was extracted from S and V samples as described in [77]. We performed ATAC-Seq on paired samples of S and V fat from nine individuals (18 samples) on the Illumina HiSeq2000. All samples were initially single-end sequenced (50 bp). We also sequenced a subset of samples with paired-end 100 bp configuration. Reads of 100 bp were trimmed to 50 bp and mapped to hg19 using BWA [78]. Following quality control, we retained 16 samples (from seven individuals with paired samples and two individuals with samples only from S) for further analysis (Supplementary Material 1: Supplementary Figure S16, Supplementary Text S2). Experimental challenges of processing a tissue with high lipid content and cell type heterogeneity led to variable yields of chromatin and subsequent sequencing depth. Given this variation, we normalized read counts across samples by scaling down to the lowest depth and pooled reads from all individuals for each tissue to explore S and V tissue characteristics. We also pooled reads from all samples (S and V) to define more general features of chromatin accessibility for adipose tissue.

##### GTEx genotypes and gene expression data

Genotypes, S and V fat RNA-Seq data from GTEx v7 were downloaded from dbGaP under accession phs000424.v7.p2. For eQTL analysis, we retained variants with MAF ≥ 0.01 in GTEx-am (~ 9 M). GTEx data were processed and analyzed using the GM analysis pipeline. Genes with RPKM ≥ 0.5 in at least 10% of individuals were used as input for DE and eQTL analysis.

### Data analysis

#### Differential expression (DE)

We compared transcriptomes across tissues (S vs V) for GM and GTEx samples. We also compared transcriptomes across populations (GM vs GTEx) for each tissue. DESeq2 [79] was used to call differentially expressed genes (DEGs) with age, sex and BMI category (individuals with or without obesity BMI >  = 30 kg/m^2^) as covariates. For each comparison, we defined DEGs at 5% False Discovery Rate (FDR) and with fold change ≥ 1.5. In GM, within each tissue, we also explored DEGs across BMI categories and sex. To probe the underlying biology of differential gene expression, we tested for enrichment of GO terms using topGO [80]. Analysis was based on gene counts using the ‘weight’ algorithm with Fisher’s exact test. Redundant GO terms were removed through REVIGO [81].

#### ATAC-Seq peak calling

We called open chromatin peaks using MACS2 [82] with flags ‘*-g hs –nomodel –nolambda –extsize 147 –keep-dup-all’*, retaining all peaks that satisfied FDR < 5% and fold enrichment > 3. Peak annotation was done using HOMER [83] and GO analysis for assigned genes was done using topGO [80].

#### eQTL mapping

Cis-eQTLs were mapped in S and V for: 1) GM (95 S and 93 V), and 2) GTEx-am (313 S and 264 V) using FastQTL (v2.184) [84]. The mapping window was defined as 1 Mb up- and down-stream of the transcription start site (TSS) for each gene. We tested for association between SNP genotypes and gene expression levels and corrected for sex, sequencing platform and the top three genotype PCs. To select the number of gene expression principal components (PCs) to include and in order to maximize discoveries for each tissue, we counted the number of eGenes (genes with at least one significant cis-eQTL) identified after incrementally increasing the number of PCs accounted for in the model from 0 to 50 or 100 by increments of ten or twenty (Supplementary Material 1: Supplementary Figure S17). An FDR threshold of < 5% was applied to identify eGenes. Autosomal eQTLs only were retained for downstream analyses. To identify eGenes with multiple independent eQTLs, we applied a forward–backward stepwise regression to learn the number of independent variants per phenotype [85]. In GM, we also explored eQTLs across individuals with or without obesity and across the sexes within each tissue. To do this, we applied linear regression with Genotype × Obesity and Genotype × Sex interaction term respectively. Effects linked to obesity status were captured by adjusting our eQTL analysis for the top three PCs of gene expression (Supplementary Material 1: Supplementary Figure S18). To explicitly adjust for obesity status, we re-ran main eQTL analysis including obesity status as a covariate.

To explore allelic imbalance in gene expression, we assessed allele-specific expression (ASE) in protein-coding genes possessing a heterozygous-transcribed SNP in ≥ 7 GM individuals. SNP-level ASE data were generated for each tissue using the GATK ASEReadCounter tool [86]. Variants with UCSC 50-mer mappability < 1, simulation-based evidence of mapping bias [87] and no evidence for monoallelic expression by requiring representation of both alleles in each SNP were excluded. Only variants with ≥ 8 reads were used. To test for ASE, we performed binomial exact test and significance was set at FDR < 5% (Supplementary Material 1: Supplementary Figure S19). Functional characterization of ASE sites and assignment of SIFT and Polyphen2 scores for pathogenicity prediction for missense variants was done through Ensembl VEP [88].

#### Properties of eQTLs

We calculated the fixation index (Fst) to directly measure allele frequency differentiation across populations using Plink [69]. To functionally characterize eQTLs, we tested whether they are enriched in adipose tissue annotations including promoters, enhancers, 15-chromatin states model (merged to 9 states) and four Chip-Seq histone marks from Roadmap Epigenomics (ID E063 V tissue), DNase hypersensitive sites (DHS) from ENCODE (V tissue), transcription factor binding sites from Remap2 (adipocytes ASC) and ATAC-Seq publicly available data (S adipocytes from [30] and S tissue from [18]). We also tested for eQTL enrichment in ATAC-Seq peaks identified in GM S and V adipose tissue. Enrichment was tested using QTLtools [76] *fenrich* with significance set at *P* < 0.05.

#### Overlap of eQTLs with GWAS signals

We downloaded the NHGRI/EBI GWAS Catalog (v1.0.2, 2021–09-05) and retained associations with *P* < 5e-8. GWAS SNPs explored were from studies in populations from all ancestries, with the majority (~ 77%) however being studies in European ancestry populations. Colocalization of GWAS variants and eQTLs was assessed using Regulatory Trait Concordance, RTC (colocalization when RTC ≥ 0.9) [89]. We summarized all tested signals into eleven broader categories using Experimental Factor Ontology (EFO) terms [90]. To uncover population differences in regulatory effects underlying disease risk, for each tissue, we explored the overlap across populations of: a) eGene-trait pairs and b) disease associated GWAS SNPs that colocalize with eQTLs.

#### Comparison of eQTLs across tissues and populations

We compared eQTLs across tissues and populations through: a) FDR-based comparisons, b) p-value enrichment analysis (replication was quantified using the pi1 statistic, [31]), and c) a linear mixed model with a Genotype × Tissue interaction term (for GM-detected eQTLs only). Specificity to a particular tissue or population was assessed by focusing on eQTLs mapping in the 5% tail of the distribution of association p-values in the replicating tissue or population. It should be noted that GTEx-am is ~ four times larger than the GM sample. Additionally, tested SNPs in GTEx-am were more (by 30%) and eQTLs were called at MAF 1%. This allows for greater overlap when comparing eQTL findings from GM to GTEX and makes our analysis stricter. Therefore, replication levels of GM findings in GTEx-am that are reported here likely reflect the upper bound of shared effects.

### Supplementary Information

**Additional file 1: Figure S1.** GM individuals map close to Italian and Spanish populations. **Figure S2.** eQTL mapping results in GM. **Figure S3.** Comparison of direction of effect for shared SNP-genes in both GM and GTEx-am, per tissue. **Figure S4.** Example of a Genotype×Sex regulatory interaction in GM S. **Figure S5.** Enrichment of GTEx-am eQTLs in adipose tissue functional annotations. **Figure S6.** eQTL plots for protein coding eGenes detected in GM only, displaying distinct expression patterns across populations. **Figure S7.** eQTL plots for non protein-coding eGenes detected in GM only, displaying distinct expression patterns across populations. **Figure S8.** Differences in expression patterns of eGenes detected in GM, but not in GTEx-am. **Figure S9.** Overlap of colocalizing GWAS SNPs between GM and GTEx-am. **Figure S10.** Secondary eQTL for *THNSL2* in GM S colocalizes with a GWAS signal associated with CRP levels.

**Additional file 2: Supplementary Table S1.** eQTLs in GM adipose tissues. **Supplementary Table S3.** GenotypexObesity significant (*p*<0.05) interactions in GM. **Supplementary Table S4.** GenotypexSex significant (*p*<0.05) interactions in GM. **Supplmentary Table S5.** eQTLs in GM tissues, when including obesity status as a covariate. **Supplementary Table S6.** Missense significant (FDR<5%) ASE SNPs identified in each tissue of GM. **Supplementary Table S7.** Replication of eQTLs across GM tissues. **Supplementary Table S8.** Detection of GM eQTLs using a linear mixed model with a Genotype×Tissue interaction term (FDR<0.05). **Supplementary Table S9.** Thirty-five eGenes identified in one GM tissue only by both p-value enrichment analysis and linear mixed model with a Genotype×Tissue interaction term. **Supplementary Table S10.** Replication of eQTLs across populations for each tissue. **Supplementary Table S11.** Thirty-two eGenes identified in both GM tissues, but not in GTEx-am. **Supplementary Table S12.** GWAS-eQTL colocalizations (RTC≥0.9) in GM for each tissue. **Supplementary Table S14.** Sharing of eGene-trait pairs in GM and GTEx-am. **Supplementary Table S15.** Expression levels of eGenes corresponding to GM specific eGene-trait pairs in GM and GTEx-am. **Supplementary Table S16.** Secondary, and not primary, eQTL colocalizations with GWAS signals in GM.

**Additional file 3: Supplementary Table S17.** DEGs by tissue in GM, GTEx-am and GTEx-sm. **Supplementary Table S18.** S vs V DEGs with discordant direction of gene expression bewteen GM and GTEx-am. **Supplementary Table S19.** DEGs by obesity status in GM tissues. **Supplementary Table S20.** DEGs by population in S and V tissue. **Supplementary Table S21.** ATAC-Seq peaks in GM.

**Additional file 4: Figure S11.** REVIGO summary for DEGs by tissue and population. **Figure S12.** REVIGO summary for 151 S vs V DEGs detected in both GM and GTEX-am, showing discordant direction of gene expression. **Figure S13.** REVIGO summary for DEGs by obesity status in GM. **Figure S14.** Chromatin accessibility landscape in GM based on ATAC-Seq data.

**Additional file 5: Supplementary Text S1.** Analysis of the GTEx-sm population sample. **Supplementary Text S2.** Open chromatin profiling (ATAC-Seq) and QC. S**upplementary Text S3.** Colocalization of GM eQTLs with cardiometabolic signals. **Supplementary Table S2.** Comparison of eQTL mapping results from GM and GTEx-sm. **Supplementary Table S13.** 117 cardiometabolic traits. **Supplementary Table S22.**  Descriptives of GM and GTEx participants. **Figure S15.** PCA on gene expression data from GM. **Figure S16.** Quality control (QC) of ATAC-Seq data. **Figure S17.** Plot of detected eGenes depending on number of expression PCs added to eQTL mapping model. **Figure S18.** Spearman’s rank correlation of first three expression principal components (PCs) with BMI (upper panel) and age (lower panel). **Figure S19.** Allele-specific expression (ASE) workflow in GM.

**Additional file 6.** Main scripts for eQTL mapping using fastQTL.

## Data Availability

Genotyping and raw RNA-Seq and ATAC-Seq data have been uploaded at the European Genome-phenome Archive (EGA), under accession number EGAS00001007126 (https://ega-archive.org/studies/EGAS00001007126). Genotypes, S and V fat RNA-Seq data from GTEx v7 are available from dbGaP under accession phs000424.v7.p2 (https://www.ncbi.nlm.nih.gov/gap/). GWAS variants used for colocalization analysis were downloaded from the NHGRI/EBI GWAS Catalog (v1.0.2, 2021–09-05, https://www.ebi.ac.uk/gwas/docs/file-downloads). Roadmap epigenomics annotations including promoters, enhancers, 15-chromatin states model (merged to 9 states) and four Chip-Seq histone marks were downloaded from Roadmap epigenomics project through data portal (https://egg2.wustl.edu/roadmap/web_portal/) (ID E063 V tissue), DNase hypersensitive sites (DHS) from the Encode project (https://www.encodeproject.org/) (V tissue) and transcription factor binding sites from Remap2 (https://remap.univ-amu.fr/download_page) (adipocytes ASC). Main scripts for eQTL mapping using fastQTL are provided in Supplementary Material 2.

## Abbreviations

1 KG: 1000 Genomes
AD: Alzheimer’s disease
ASE: Allele specific expression
ATAC-Seq: Assay for Transposase-Accessible Chromatin using sequencing
BMI: Body mass index
CRP: C-reactive protein
DE: Differential expression
DEG: Differentially expressed gene
DEPTOR: DEP Domain Containing MTOR Interacting Protein
ECT2: Epithelial Cell Transforming 2
EFO: Experimental factor ontology
eGene: Genes with at least one cis-eQTL
eQTL: Expression quantitative trait locus
FDR: False discovery rate
FMO2: Flavin Containing Dimethylaniline Monoxygenase 2
Fst: Fixation index
GM: Greek Metabolic
GO: Gene ontology
GTEx: Genotype-Tissue Expression
GTEx-am: GTEx-ancestry-matched
GTEx-sm: GTEx-size-matched
GWAS: Genome-wide association study
HBB: Hemoglobin Subunit Beta
HBG2: Hemoglobin Subunit Gamma 2
HWE: Hardy–Weinberg equilibrium
IBD: Inflammatory bowel disease
IBS: Iberian
IMMT: Inner Membrane Mitochondrial Protein
LD: Linkage disequilibrium
LDLR: Low Density Lipoprotein Receptor
LUZP6: Leucine Zipper Protein 6
MA: Minor allele
MAF: Minor allele frequency
MAN2A1: Mannosidase Alpha Class 2A Member 1
MIR21: MicroRNA 21
NTRK2: Neurotrophic Receptor Tyrosine Kinase 2
PCA: Principal component analysis
PCDHB13: Protocadherin Beta 13
PCs: Principal components
PM: Particulate matter
PLTP: Phospholipid Transfer Protein
RIN: RNA integrity number
RPKM: Reads per kilobase per million
RTC: Regulatory trait concordance
SELPLG: Selectin P Ligand
SET: SET Nuclear Proto-Oncogene
SNP: Single nucleotide polymorphism
SNX: Sorting nexin
SNX33: Sorting Nexin 33
ST7: Suppression Of Tumorigenicity 7
SULT1A2: Sulfotransferase Family 1A Member 2
THNSL2: Threonine Synthase Like 2
TSI: Tuscan
TSS: Transcription start site
WHR: Waist-to-hip ratio
