# Supplementary material for: Identifying novel regulatory effects for clinically relevant genes through the study of the Greek population
Source: BMC Genomics. 2023 Aug 5;24:442. doi: 10.1186/s12864-023-09532-w (PMC10403965; doi:10.1186/s12864-023-09532-w)
Supplement: Supplementary file 1 — Additional file 1: Figure S1. GM individuals map close to Italian and Spanish populations. Figure S2. eQTL mapping results in GM. Figure S3. Comparison of direction of effect for shared SNP-genes in both GM and GTEx-am, per tissue. Figure S4. Example of a Genotype×Sex regulatory interaction in GM S. Figure S5. Enrichment of GTEx-am eQTLs in adipose tissue functional annotations. Figure S6. eQTL plots for protein coding eGenes detected in GM only, displaying distinct expression patterns across populations. Figure S7. eQTL plots for non protein-coding eGenes detected in GM only, displaying distinct expression patterns across populations. Figure S8. Differences in expression patterns of eGenes detected in GM, but not in GTEx-am. Figure S9. Overlap of colocalizing GWAS SNPs between GM and GTEx-am. Figure S10. Secondary eQTL for THNSL2 in GM S colocalizes with a GWAS signal associated with CRP levels. [file 12864_2023_9532_MOESM1_ESM.docx]

**Additional File 1**

Supplementary Figures S1-S10.

**
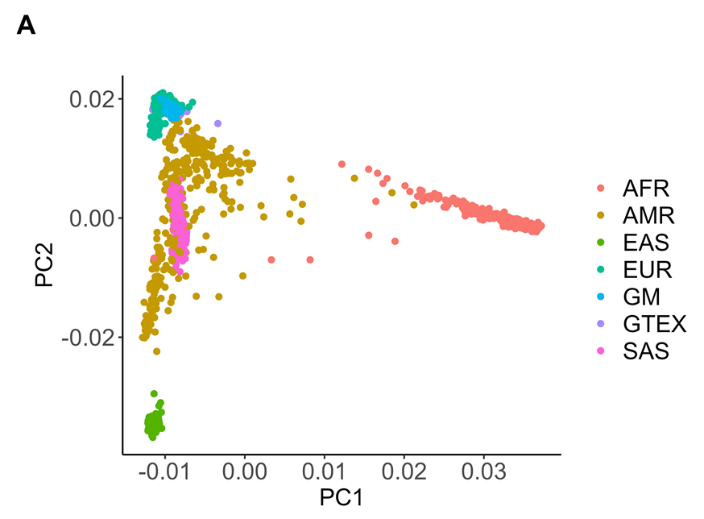

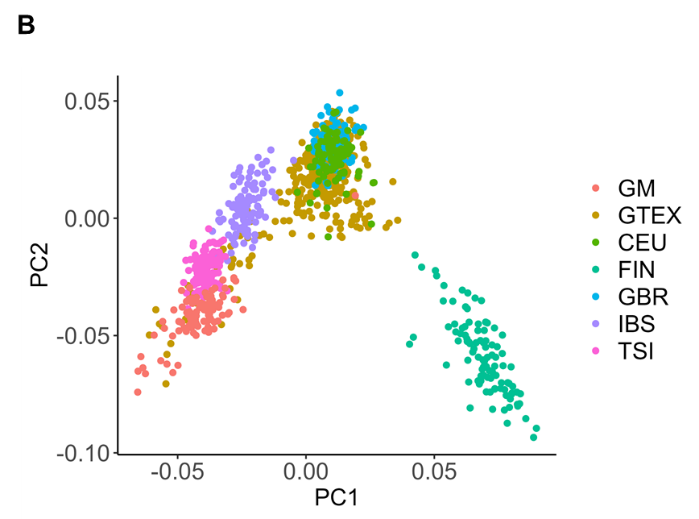
**

**Figure S1. GM individuals map close to Italian and Spanish populations.** Principal component analysis (PCA) of genotype data (MAF≥0.05) reveals that GM individuals map close to: A) the 1KG EUR superpopulation, and B) the IBS and TSI 1Kg populations. Abbreviations: GM, Greek Metabolic study; GTEX, GTEx-ancestry-matched; CEU, Central European; FIN, Finnish; GBR, British; IBS, Iberian; TSI, Tuscan, AFR, African; AMR, Admixed American; EAS, East Asian; EUR, European; SAS, South Asian.

**
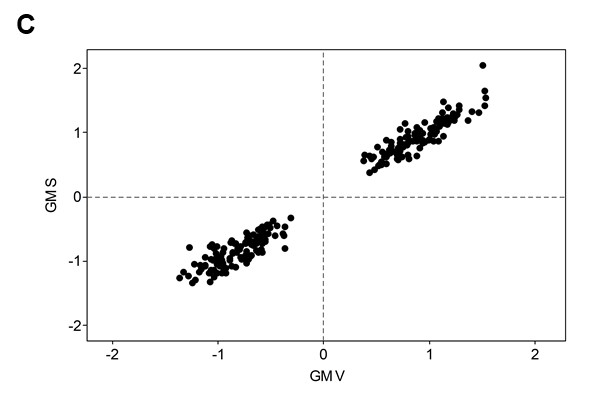

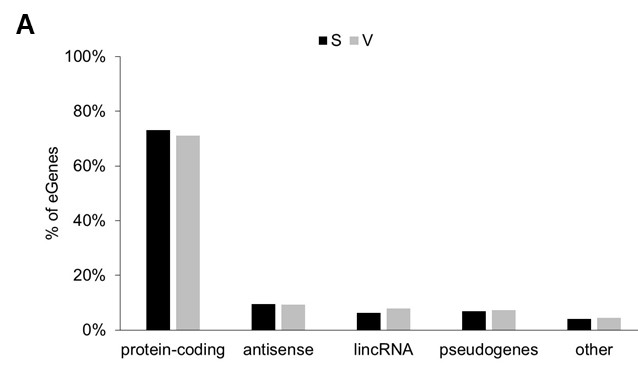

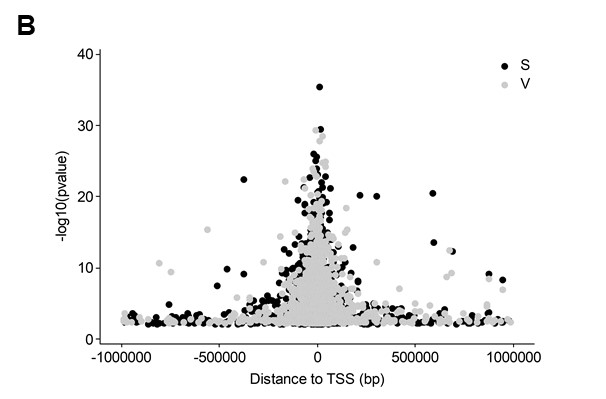

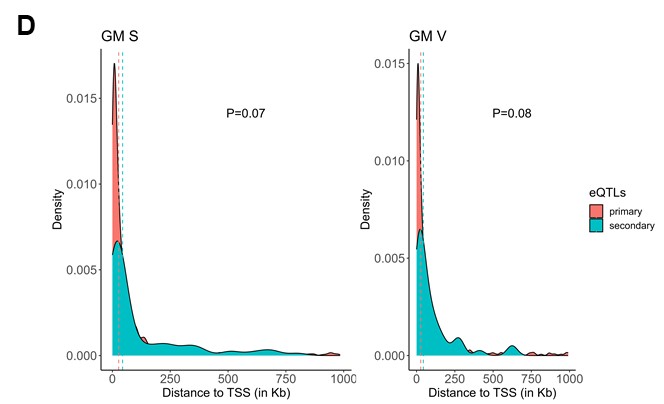
**

**Figure S2. eQTL mapping results in GM.** A) eGene biotypes. B) Clustering of eQTLs around TSS. C) Direction of effect of shared SNP-genes (232) between S and V adipose tissue (R=0.988, P<1e^-04^). D) Density plot showing the distance from the lead eQTL variant to the TSS. Dashed lines represent the median distance to the TSS for primary (red) and secondary (blue) eQTLs. (M-W test p-values between primary and secondary eQTLs are shown). GM: Greek Metabolic; S: subcutaneous; V: visceral.


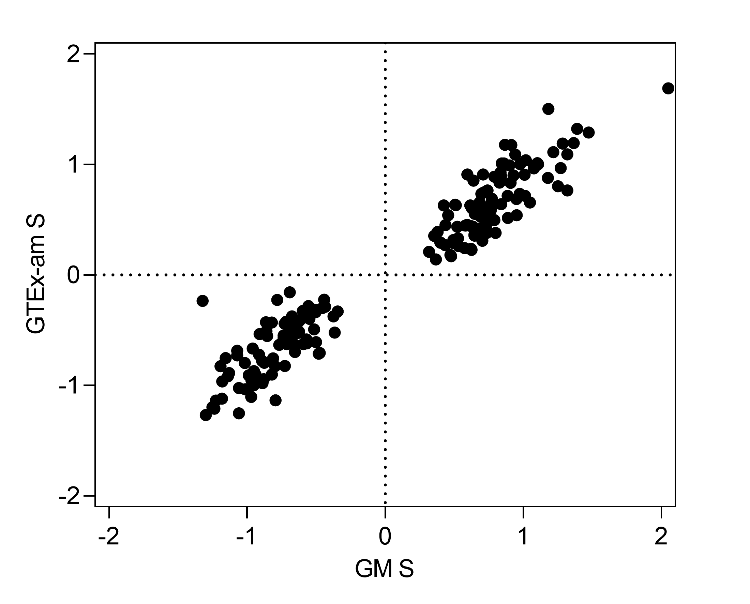

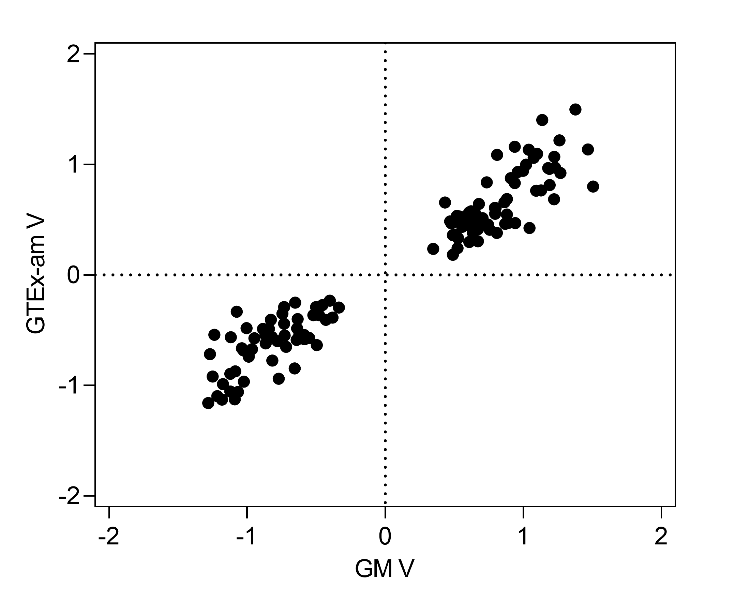


**Figure S3.** **Comparison of direction of effect for shared SNP-genes in both GM and GTEx-am, per tissue**. Shared SNP-genes were 178 and 123 in S and V, respectively. GM: Greek Metabolic; GTEx-am: GTEx-ancestry-matched; S: subcutaneous; V: visceral.


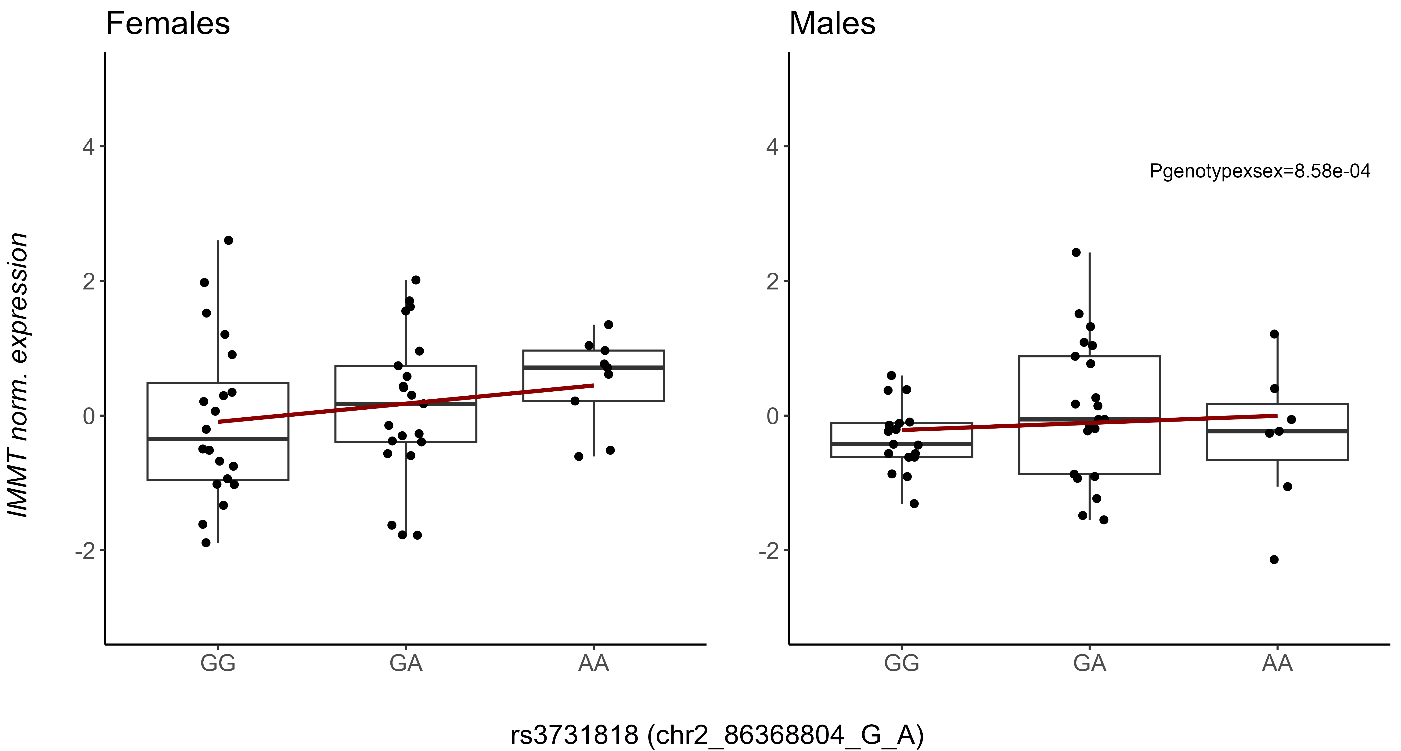


**Figure S4. Example of a Genotype×Sex regulatory interaction in GM S.** *IMMT*; Inner Membrane Mitochondrial Protein. P value is from linear model using interaction term Genotype×Obesity. Genomic coordinates of rs3731818 are from GRCh37 (hg19) build.





**Figure S5. Enrichment of GTEx-am eQTLs in adipose tissue functional annotations.** Enrichment of GTEx-am eQTLs in adipose tissue functional annotations is shown as estimated odds ratios and 95% confidence intervals on the x axis for each annotation category on the y axis. Odds ratios greater than 1 indicate an enrichment of QTL variants in the given functional annotations, while odds ratios less than 1 indicate a depletion. Significant odds ratios are shown as filled circles or squares (P < 0.05). Cannon et al: ref 18; Allum et al: ref 30. GTEx-am: GTEx-ancestry-matched; S: subcutaneous; V: visceral.


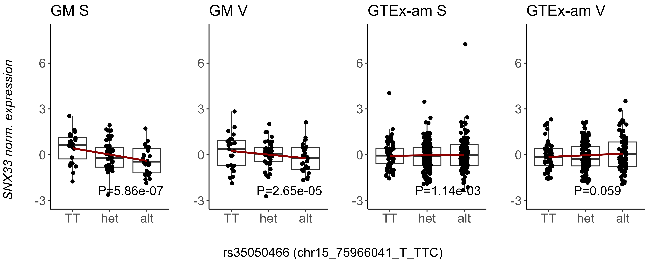

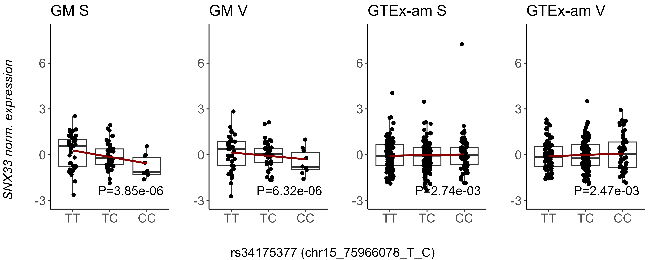

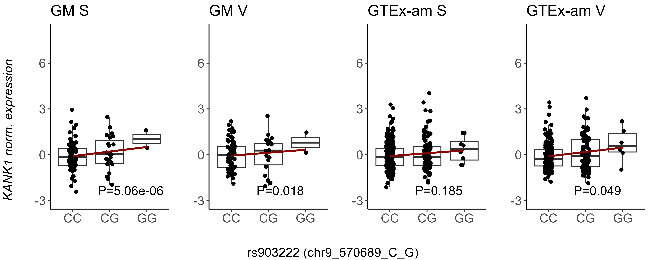


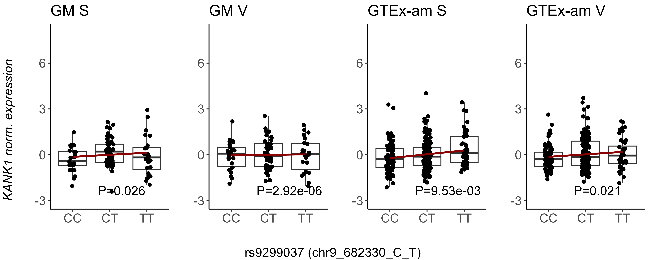

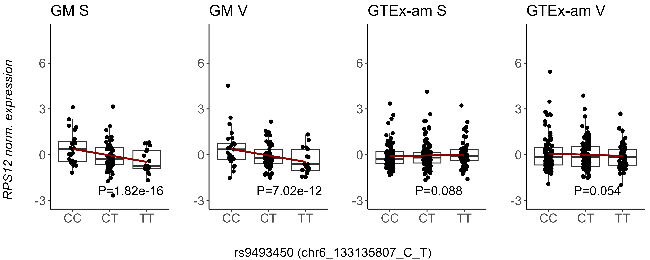

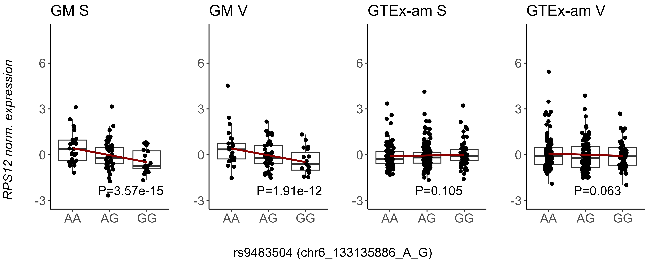


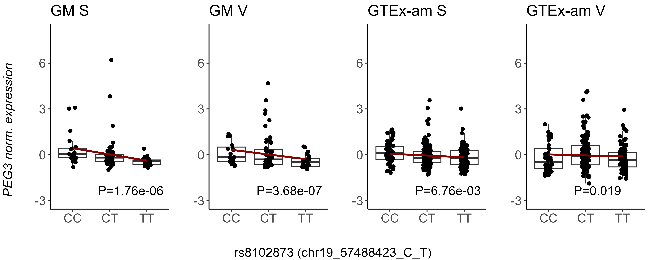


**Figure S6. eQTL plots for protein coding eGenes detected in GM only, displaying distinct expression patterns across populations.** Genes were identified as eGenes in both GM S and V tissue, but not in GTEx-am. The associated eQTLs differ between S and V tissue of GM, apart from *PEG3*-rs8102873 eQTL that is shared. P-values represent association nominal p-values from nominal pass of fastQTL. Genomic coordinates of eQTLs are from GRCh37 (hg19) build. S: subcutaneous; V: visceral; GM: Greek Metabolic; GTEx-am: GTEx-ancestry matched; *SNX33*: Sorting Nexin 33; *PEG3*: Paternally Expressed 3; *KANK1*: KN Motif And Ankyrin Repeat Domains 1; *RPS12*: Ribosomal Protein S12.


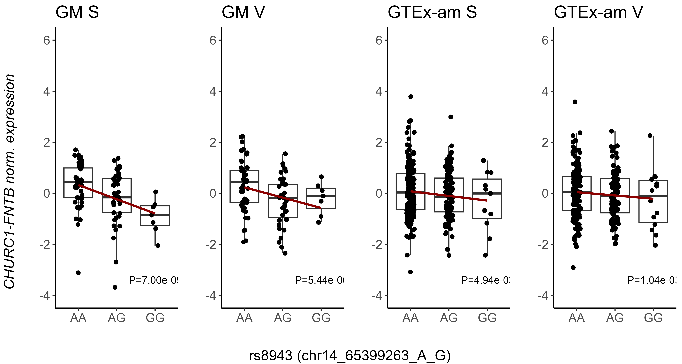

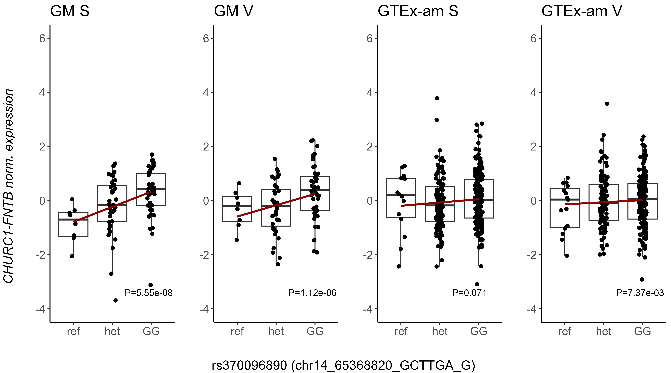

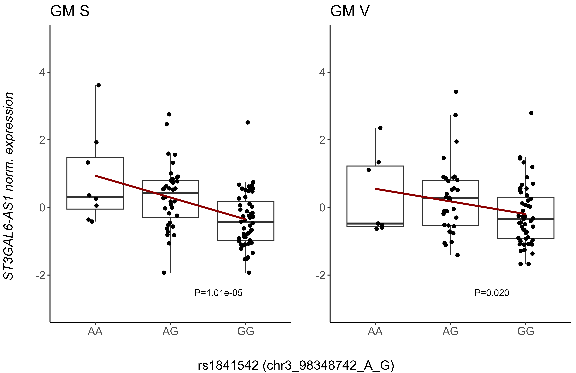


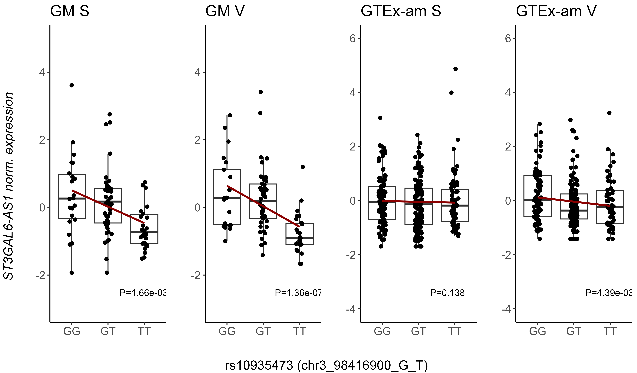


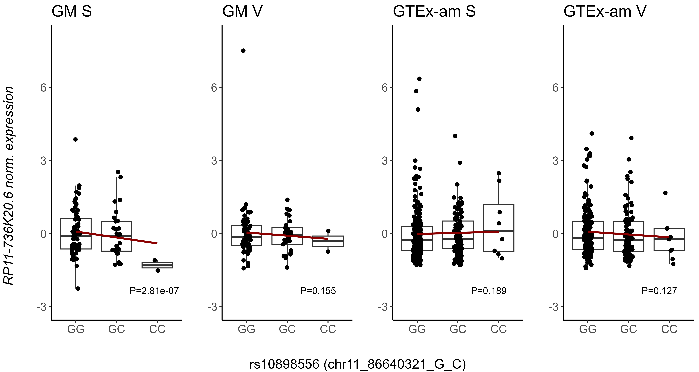

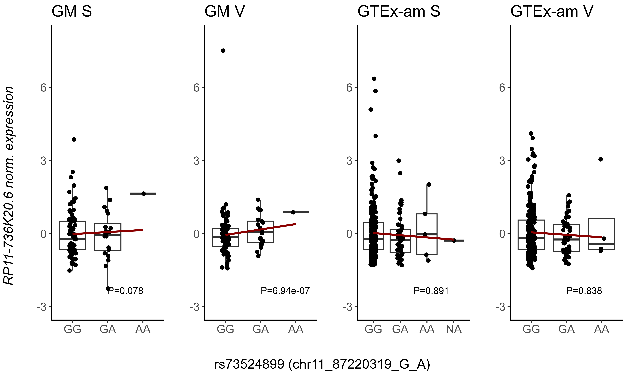


**Figure S7. eQTL plots for non protein-coding eGenes detected in GM only, displaying distinct expression patterns across populations.** Genes were identified as eGenes in GM, but not GTEx-am. The associated eQTLs differ between S and V tissue of GM. P-values represent association nominal p-values. Genomic coordinates of eQTLs are from GRCh37 (hg19) build. GM: Greek Metabolic; GTEx-am: GTEx-ancestry-matched: S: subcutaneous; V: visceral.




















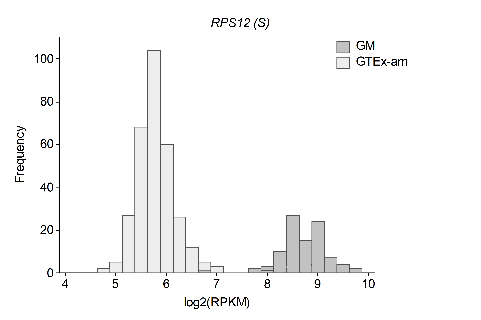






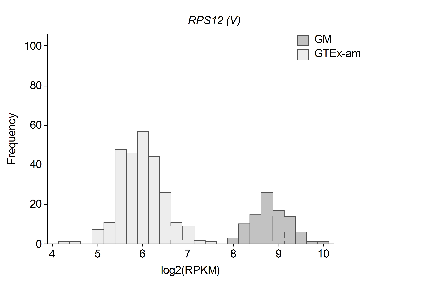




**Figure S8. Differences in expression patterns of eGenes detected in GM, but not in GTEx-am.** We hypothesize that the observed differences may arise in part due to environmental factors. Distribution of expression patterns differ in all cases (P<1e-05; M-W test), apart from PEG3 (V), P=0.4939.


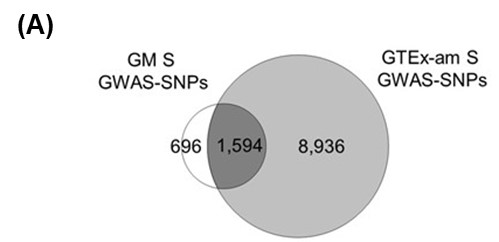

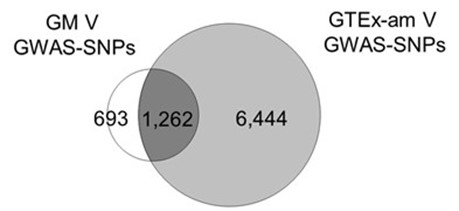


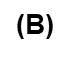


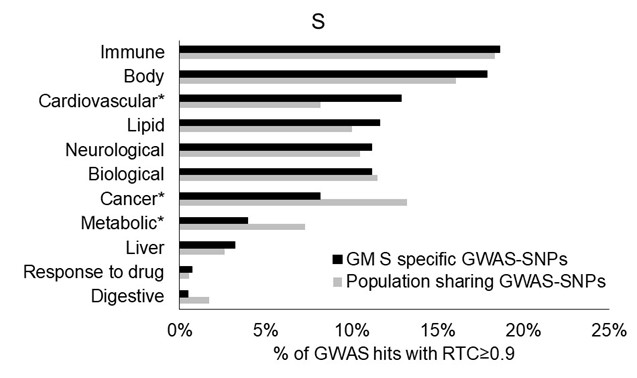

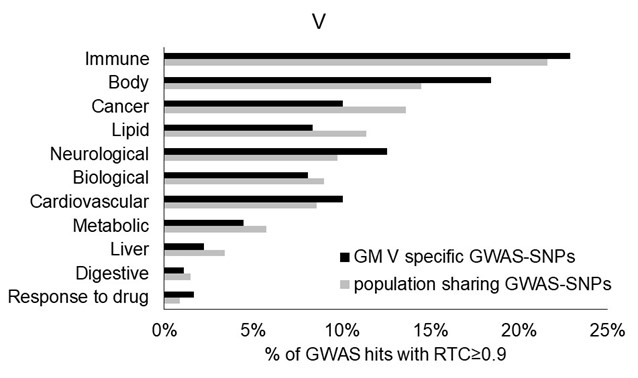


**Figure S9. Overlap of colocalizing GWAS SNPs between GM and GTEx-am.** (A) Venn diagram of GWAS SNPs across GM and GTEx-am for each tissue. (B) Frequency in % of EFO categories related to GWAS signals that are specific to GM or population sharing. Statistical significance was tested using Fisher’s test (* for P<0.05). GM: Greek Metabolic; GTEx-am: GTEx-ancestry-matched: S: subcutaneous; V: visceral.


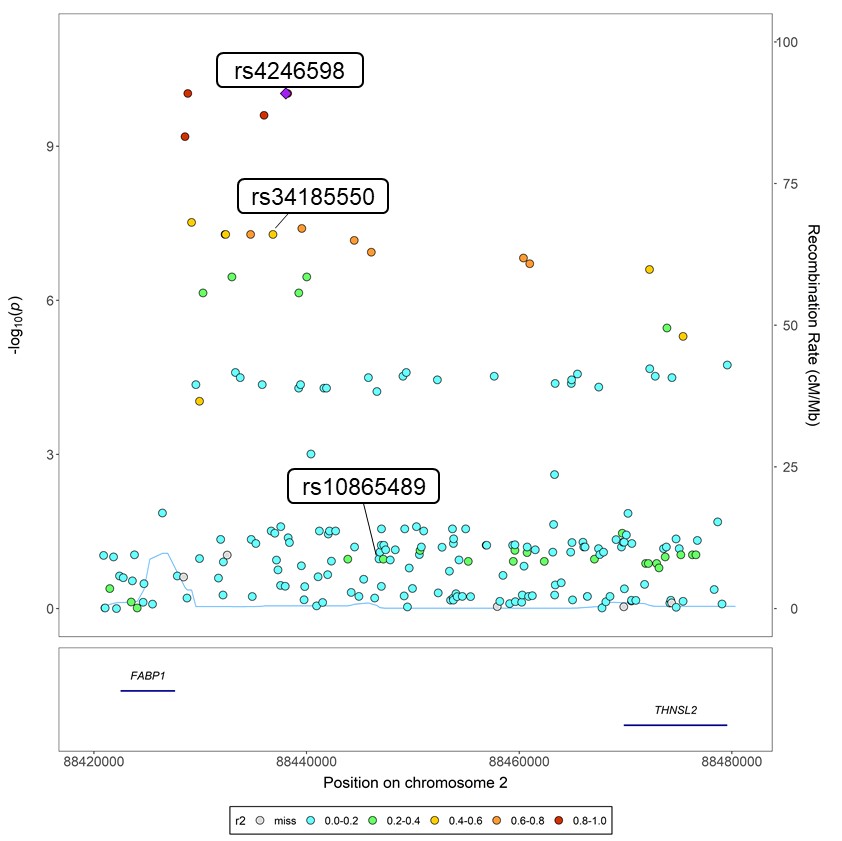


**Figure S10. Secondary eQTL for *THNSL2* in GM S colocalizes with a GWAS signal associated with CRP levels.** A regional association plot is presented. The GWAS variant associated with CRP levels, rs4246598 (top), colocalizes with the secondary eQTL rs34185550 (middle), but not with the primary eQTL rs10865489 (bottom). Variants are colored by the strength of their LD with the GWAS variant (diamond), with darker colors indicating stronger LD. T*HNSL2:* Threonine Synthase Like 2.
