# Supplementary material for: Identifying novel regulatory effects for clinically relevant genes through the study of the Greek population
Source: BMC Genomics. 2023 Aug 5;24:442. doi: 10.1186/s12864-023-09532-w (PMC10403965; doi:10.1186/s12864-023-09532-w)
Supplement: Supplementary file 4 — Additional file 4: Figure S11. REVIGO summary for DEGs by tissue and population. Figure S12. REVIGO summary for 151 S vs V DEGs detected in both GM and GTEX-am, showing discordant direction of gene expression. Figure S13. REVIGO summary for DEGs by obesity status in GM. Figure S14. Chromatin accessibility landscape in GM based on ATAC-Seq data. [file 12864_2023_9532_MOESM4_ESM.docx]

**Additional File 4**

Supplementary Figures S11-S14


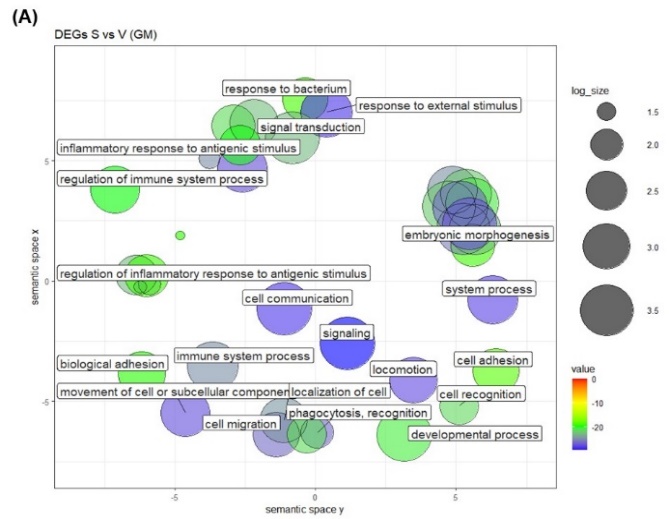

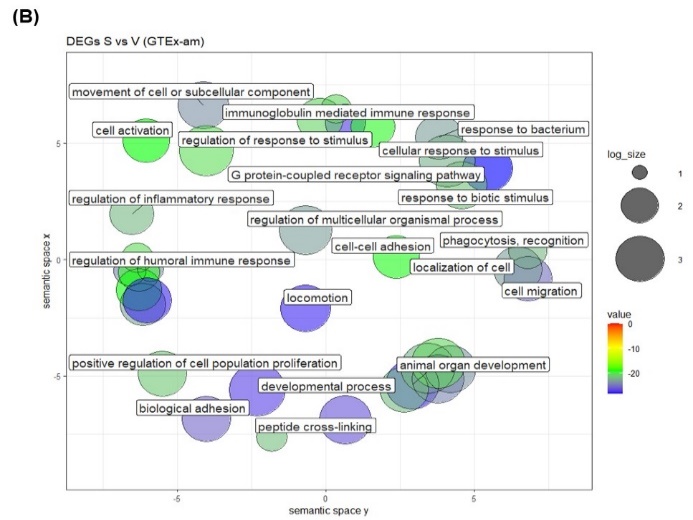


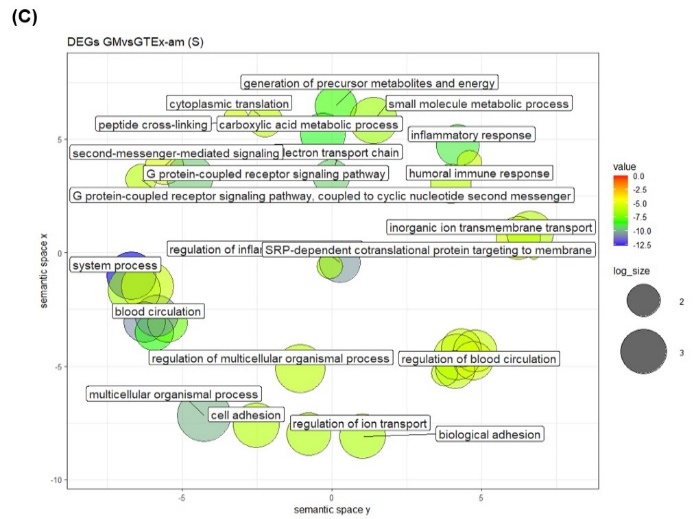

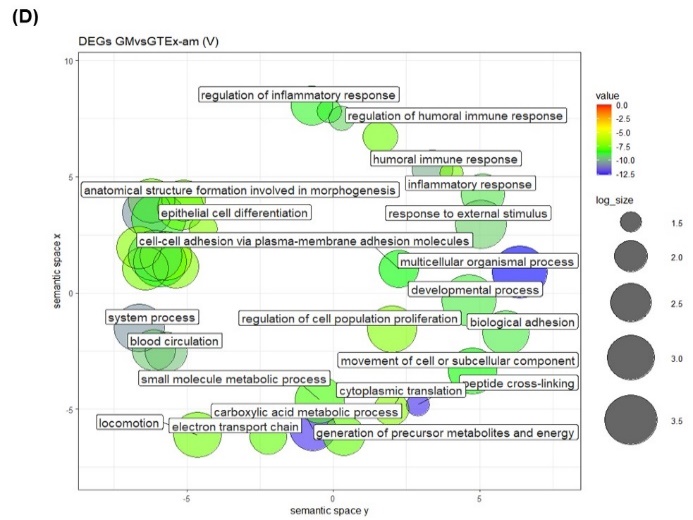


**Figure S11. REVIGO summary for DEGs by tissue and population.** (A) 2,979 S vs V DEGs in GM. (B) 5,320 S vs V DEGs in GTEx-am. (C) 12,459 GM vs GTEx-am DEGs in S. (D) 11,652 GM vs GTEx-am DEGs in V. Top 50 statistically significant GO BP terms were used as input for REVIGO.


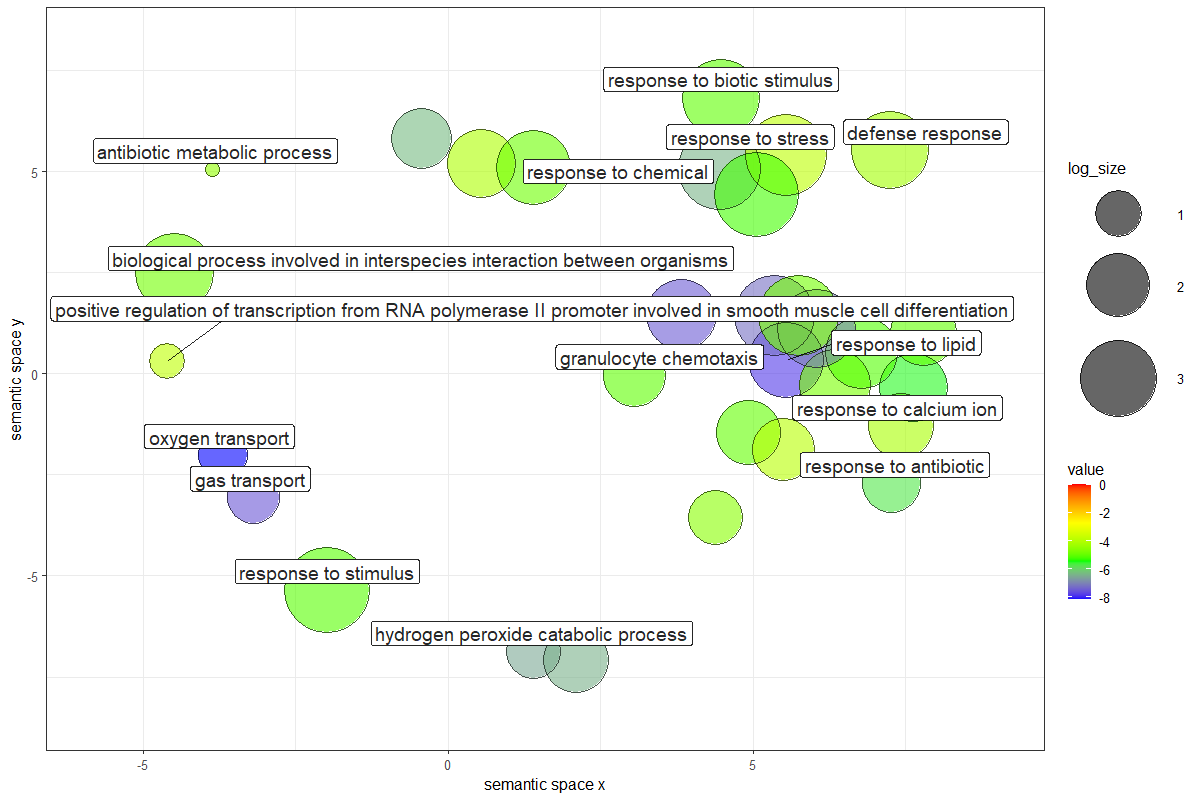


**Figure S12. REVIGO summary for 151 S vs V DEGs detected in both GM and GTEX-am, showing discordant direction of gene expression.** Top 50 statistically significant GO BP terms were used as input for REVIGO.


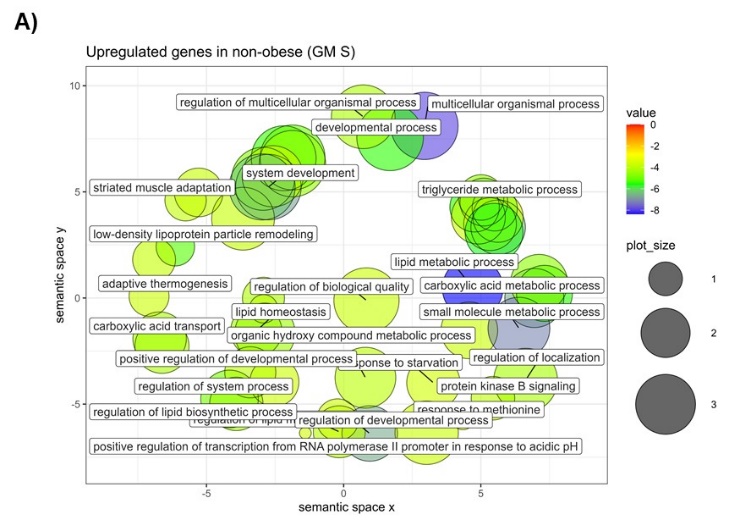

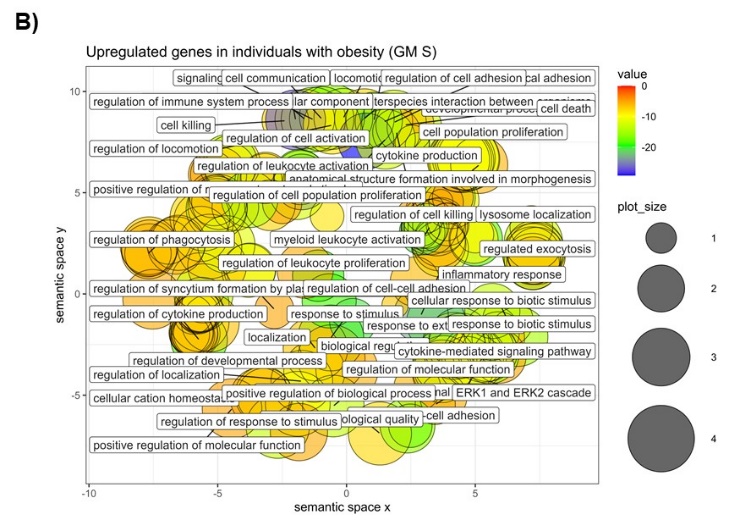


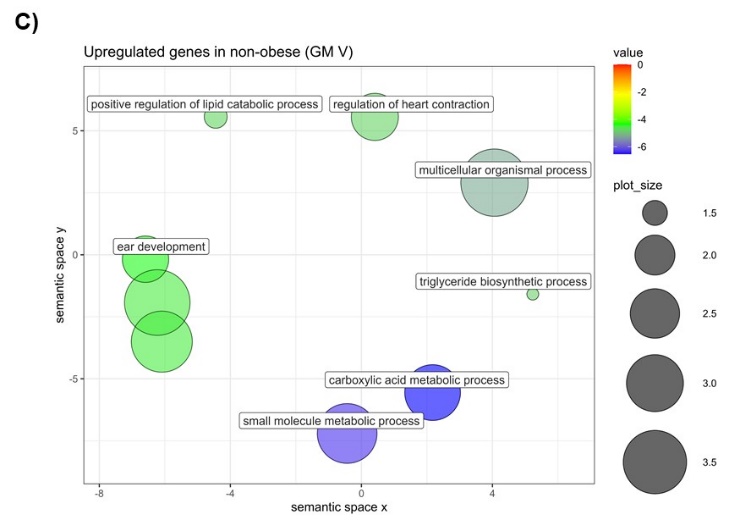

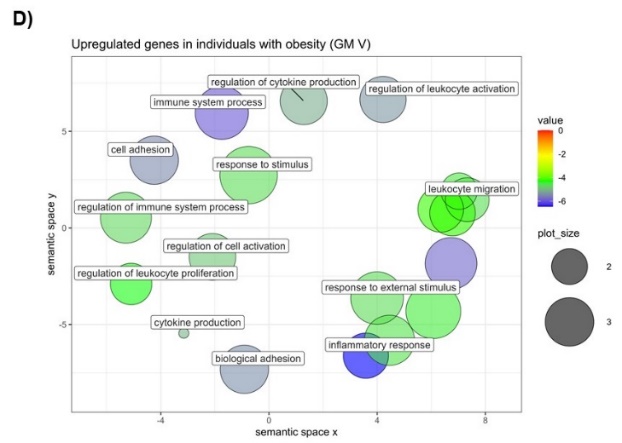


**Figure S13. REVIGO summary for DEGs by obesity status in GM.** In GM S, (A) 352 upregulated genes in non-obese and (B) 702 upregulated genes in individuals with obesity. In GM V, (C) 166 upregulated genes in non-obese and (D) 263 upregulated genes in individuals with obesity. Top 50 statistically significant GO BP terms were used as input for REVIGO.


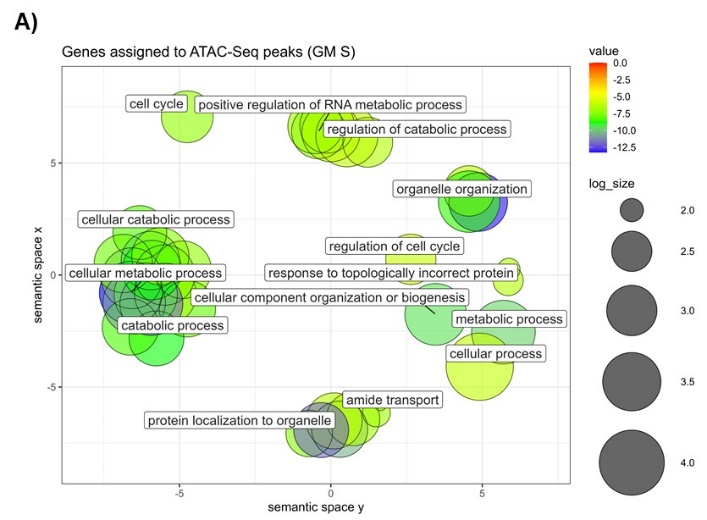

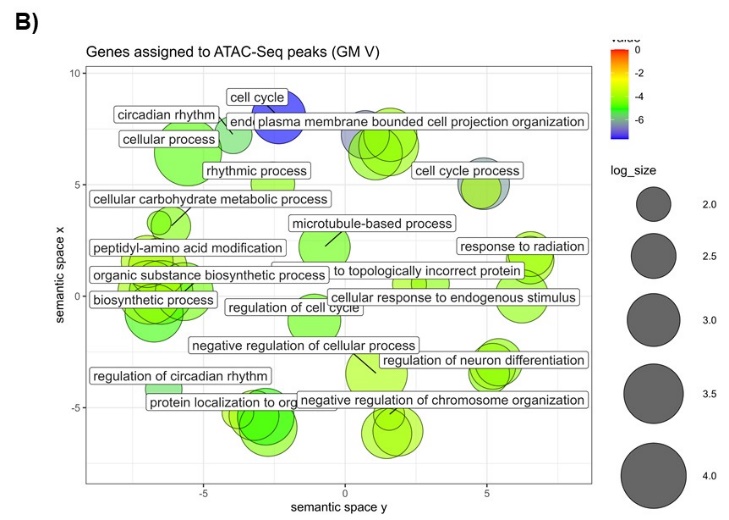


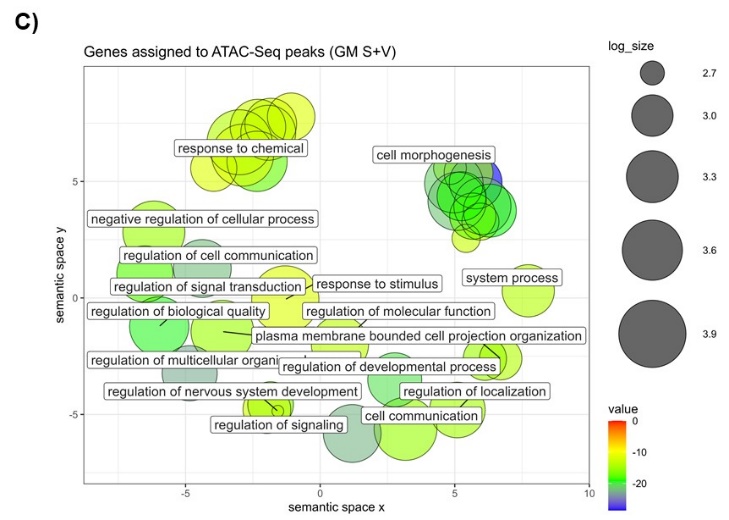


**Figure S14. Chromatin accessibility landscape in GM based on ATAC-Seq data.** REVIGO summary of GO_BP terms for genes assigned to (A) 16,907 ATAC-Seq peaks in S, (B) 14,700 ATAC-Seq peaks in V and (C) 121,673 global (S+V) adipose peaks. Top 50 statistically significant GO BP terms were used as input for REVIGO.
