# Supplementary material for: Identifying novel regulatory effects for clinically relevant genes through the study of the Greek population
Source: BMC Genomics. 2023 Aug 5;24:442. doi: 10.1186/s12864-023-09532-w (PMC10403965; doi:10.1186/s12864-023-09532-w)
Supplement: Supplementary file 5 — Additional file 5: Supplementary Text S1. Analysis of the GTEx-sm population sample. Supplementary Text S2. Open chromatin profiling (ATAC-Seq) and QC. Supplementary Text S3. Colocalization of GM eQTLs with cardiometabolic signals. Supplementary Table S2. Comparison of eQTL mapping results from GM and GTEx-sm. Supplementary Table S13. 117 cardiometabolic traits. Supplementary Table S22. Descriptives of GM and GTEx participants. Figure S15. PCA on gene expression data from GM. Figure S16. Quality control (QC) of ATAC-Seq data. Figure S17. Plot of detected eGenes depending on number of expression PCs added to eQTL mapping model. Figure S18. Spearman’s rank correlation of first three expression principal components (PCs) with BMI (upper panel) and age (lower panel). Figure S19. Allele-specific expression (ASE) workflow in GM. [file 12864_2023_9532_MOESM5_ESM.docx]

**Supplementary Material**

Supplementary Texts S1-S3

Supplementary Tables S2, S13, S22

Supplementary Figures S15-S19

**Supplementary Text S1**

Analysis of the GTEx-sm population sample

To match for ancestry, size and phenotypic characteristics (age, sex ratio, % obesity), we performed all eQTL analyses in the GTEx-sm population sample. For the eQTL mapping, we retained variants with MAF≥0.05 in GTEx-sm (~6.2M) accounting for differences in sample size (compared to MAF>=0.01 in GTEX-am). Cis-eQTLs were mapped in S and V for 95 and 93 samples respectively. We detected similar levels of eGenes (1,692 in S and 1,250 in V; 807 shared) as in GM **Supplementary Table S2**). Comparison of GM to GTEx-sm eQTLs yielded replication rates of 82% in S and 86% in V. Similar to GM levels of GWAS-eQTL colocalization were observed in GTEx-sm, with 554 (46.6% of tested) and 427 (of 875 tested) eQTLs colocalizing with GWAS SNPs in S and V, respectively. We also performed differential expression in GTEx-sm and found similar to GTEx-am number of DEGs (5,283 genes) (**Additional File 2: Supplementary Table S17).**

**Supplementary Text S2**

Open chromatin profiling (ATAC-Seq) and QC

We performed ATAC-Seq on paired samples of S and V from nine individuals (18 samples) on the Illumina HiSeq2000 by using single-end 50bp sequencing mode (SEx50). We also sequenced a subset of samples with paired-end mode (PEx100), resulting in 28 sequenced samples. After trimming 100bp to 50bp reads and mapping to hg19 using BWA, we applied QC analysis. We excluded two samples that had low library complexity, as indicated by NRF (non-redundant fraction) and PBC1 (PCR Bottlenecking Coefficient 1) or low signal-to-noise ratio. For the 14 samples sequenced in both single- or paired-end mode, BAM files were merged, resulting in a total number of 16 samples (seven individuals with paired samples and two individuals only from S), retained for further analysis.

**Supplementary Text S3**

*Colocalization of GM eQTLs with cardiometabolic signals*

We filtered the GM GWAS-eQTL colocalizations for terms related to cardiometabolic traits (117 traits in total, S Table 13). We found that 234 (36.1%) and 195 (35.3%) eQTLs colocalize with 471 and 368 GWAS signals, in S and V respectively.

| **Supplementary Table S2. Comparison of eQTL mapping results from GM and GTEx-sm** | | | |
| --- | --- | --- | --- |
| **Across tissues (S vs. V)** | | | |
| **GM** | **eGenes** | **eQTLs** | **eQTL-eGene pairs** |
| *S* | 1,930 | 1,847 | 1,930 |
| *V* | 1,515 | 1,448 | 1,515 |
| *overlapping* | 1,047 | 237 | 232 |
| **GTEx-sm** | **eGenes** | **eQTLs** | **eQTL-eGene pairs** |
| *S* | 1,692 | 1,587 | 1,692 |
| *V* | 1,250 | 1,168 | 1,250 |
| *overlapping* | 807 | 142 | 138 |
| **Across populations (GM vs. GTEx-sm)** | | | |
| **S** | **eGenes** | **eQTLs** | **eQTL-eGene pairs** |
| *GM* | 1,930 | 1,847 | 1,930 |
| *GTEx-sm* | 1,692 | 1,587 | 1,692 |
| *overlapping* | 881 | 94 | 92 |
| **V** | **eGenes** | **eQTLs** | **eQTL-eGene pairs** |
| *GM* | 1,515 | 1,448 | 1,515 |
| *GTEx-sm* | 1,250 | 1,168 | 1,250 |
| *overlapping* | 690 | 71 | 72 |

**Supplementary Table S13. 117 cardiometabolic traits**

| Abdominal aortic aneurysm | Metabolic syndrome |
| --- | --- |
| Adiponectin levels | Metabolic traits |
| Adult onset asthma or type 2 diabetes | Metabolite levels (lipid measures) |
| Albumin-globulin ratio | Metabolite levels (lipoprotein measures) |
| Angiopoietin-1 receptor levels | Myocardial infarction |
| Anorexia nervosa | Non-lobar intracerebral hemorrhage (MTAG) |
| Apolipoprotein A1 levels | Obesity |
| Apolipoprotein B levels | Obesity-related traits |
| Appendicular lean mass | Peripheral artery disease |
| Atrial fibrillation | Phosphatidylinositol levels |
| Behcet's disease | Phospholipid levels (plasma) |
| Blood metabolite levels | Plasma homocysteine levels (post-methionine load test) |
| Blood pressure | Plasma omega-6 polyunsaturated fatty acid levels |
| Blood protein levels in cardiovascular risk | Plasma plasminogen activator levels |
| Body fat distribution | Platelet reactivity measurement (collagen-epinephrine) |
| Body fat percentage | Postprandial triglyceride levels |
| Body mass index | PR interval |
| Cardiometabolic and hematological traits | Protein levels in obesity |
| Cardiovascular disease | Pulse pressure |
| Chemerin levels | QRS complex (Cornell) |
| Total cholesterol levels | QRS duration |
| Chronic obstructive pulmonary disease or high blood pressure (pleiotropy) | QT dynamics during recovery from exercise |
| Chronic obstructive pulmonary disease or resting heart rate (pleiotropy) | QT interval |
| Circulating chemerin levels | Random C-peptide levels in type I diabetes |
| Congenital left-sided heart lesions | Renal underexcretion gout |
| Coronary artery disease | Serum uric acid levels |
| Coronary heart disease | Soluble ICAM-1 |
| C-reactive protein levels or HDL-cholesterol levels (pleiotropy) | Soluble levels of adhesion molecules |
| Creatine kinase levels | Sphingolipid levels |
| Dilated cardiomyopathy (MTAG) | Stroke |
| Electrocardiogram morphology (amplitude at temporal datapoints) | Systemic sclerosis |
| Electrocardiographic traits (multivariate) | Takayasu arteritis |
| Eosinophilic granulomatosis with polyangiitis | Thrombosis |
| Eosinophilic granulomatosis with polyangiitis (MPO-ANCA positive) | Tissue-type plasminogen activator levels |
| Fasting blood glucose | TPE interval (resting) |
| Fasting blood insulin (BMI interaction) | Triglyceride levels |
| Fat-free mass | Type 1 diabetes |
| Fulminant type 1 diabetes | Type 2 diabetes |
| Gastric parietal cell autoantibody levels in type 1 diabetes | Urate levels |
| Giant cell arteritis | Venous thromboembolism |
| Glycated hemoglobin levels | Waist circumference |
| Glycerophospholipid levels | Waist circumference adjusted for BMI |
| Haemorrhoidal disease | Waist-hip ratio |
| HDL cholesterol | Waist-to-hip ratio adjusted for BMI |
| Heart failure | Weight |
| Heart rate | Brugada syndrome |
| Hemostatic factors and hematological phenotypes | Chronic obstructive pulmonary disease or coronary artery disease (pleiotropy) |
| Hip circumference | C-reactive protein levels or triglyceride levels (pleiotropy) |
| Hip circumference adjusted for BMI | Homocysteine levels |
| Hypertension | Hypertrophic cardiomyopathy (MTAG) |
| Hypertrophic cardiomyopathy | Lipid traits (pleiotropy) (HIPO component 1) |
| Insulin disposition index | Lipoprotein-associated phospholipase A2 activity and mass |
| Insulin-like growth factors | Migraine |
| Ischemic stroke | Mitral valve prolapse |
| Lacunar stroke | Myocardial infarction (early onset) |
| Latent autoimmune diabetes vs type 1 diabetes | Palmitoleic acid (16:1n-7) levels |
| Latent autoimmune diabetes vs type 2 diabetes | Spontaneous coronary artery dissection |
| LDL cholesterol |  |
| Lipid metabolism phenotypes |  |
| Lipid traits |  |

| **Supplementary Table S22. Descriptives of GM and GTEx participants** | | |  |
| --- | --- | --- | --- |
|  | **GM^a^** | **GTEx-am^b^** | **GTEx-sm^c^** |
| **N of individuals** | 106 | 391 | 158 |
| **Age (yrs)** | 53.79±17.16 | 52.96±17.16 | 52.6±12.90 |
| **BMI (Kg/m^2^)** | 33.24±10.89 | 27.28±4.10 | 28.51±4.59 |
| **% females** | 54 (50.9%) | 143 (36.6%) | 77 (48.7%) |
| **% with obesity** | 50 (47.2%) | 107 (27.4%) | 68 (43.0%) |
| **S tissue samples used in analyses** |  |  |  |
| *Differential expression (DE)* | 102 | 313 | 95 |
| *eQTLs mapping* | 95 | 264 | 93 |
| *ATAC-Seq* | 9 | - | - |
| **V tissue samples used in analyses** |  |  |  |
| *Differential expression (DE)* | 99 | 313 | 95 |
| *eQTLs mapping* | 93 | 264 | 93 |
| *ATAC-Seq* | 9 | - | - |

^a^ GM, Greek Metabolic. ^b^ GTEx-am, GTEX-ancestry-matched. ^c^ GTEx-sm, GTEX-size-matched. Numeric variables are represented as mean±SD. Categorical variables are shown as numbers (%)


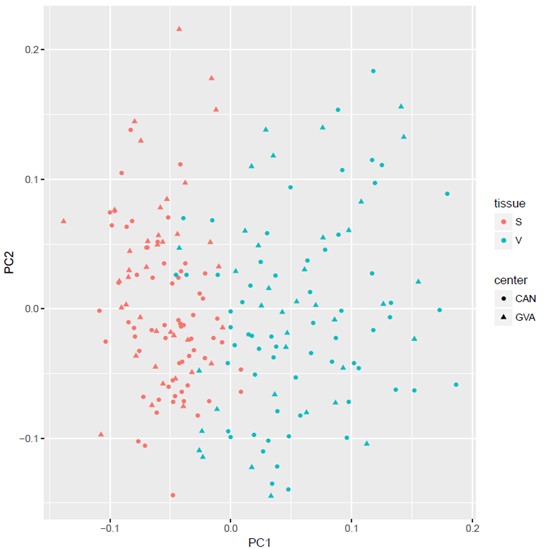


**Figure S15.** PCA on gene expression data from GM. Two clusters exist as GM study explored two discrete tissues (S and V). S: subcutaneous; V: visceral; CAN: Canada; GVA: Geneva.


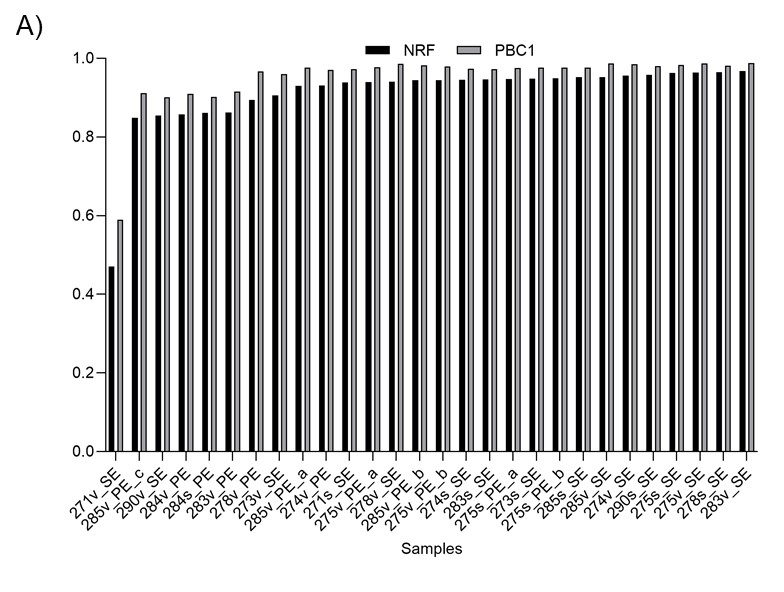


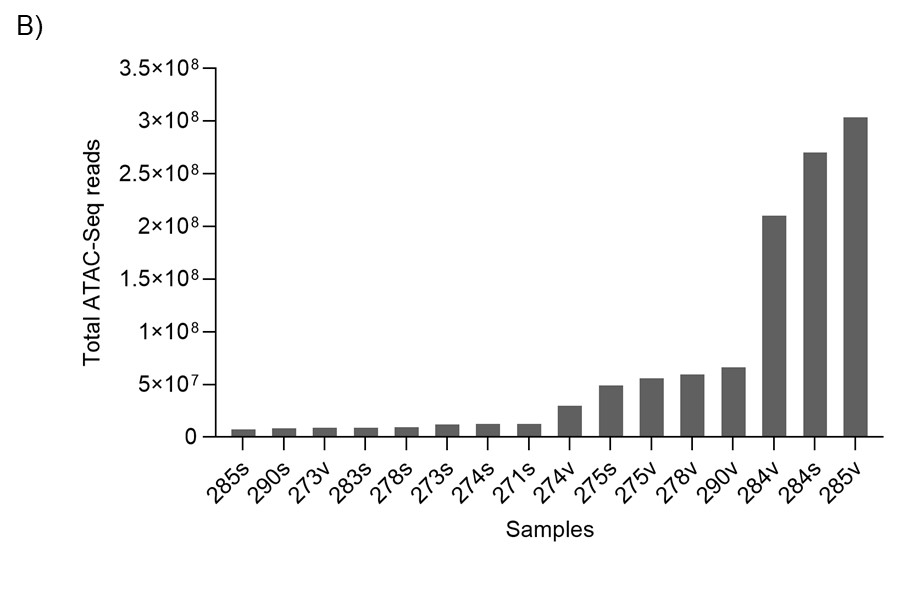


**Figure S16. Quality control (QC) of ATAC-Seq data.** (A) NRF (non-redundant fraction) and PBC1 (PCR Bottlenecking Coefficient 1) were tested for measuring library complexity (values >0.7, based on ENCODE standards, are considered acceptable). (B) Number of total reads for 16 libraries retained after QC.




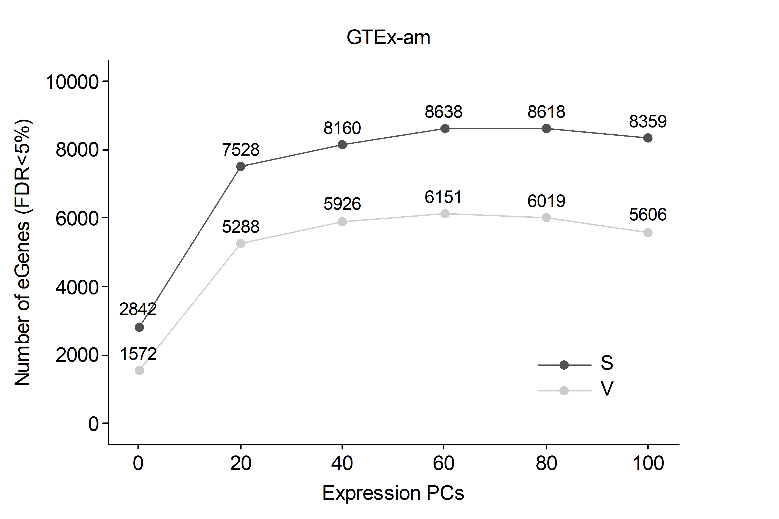





**Figure S17. Plot of detected eGenes depending on number of expression PCs added to eQTL mapping model.** We tested different numbers of expression PCs as covariates in eQTL mapping in order to maximize power for eGene discovery. Using the threshold of FDR<5%, we found that 20, 60 and 20 expression PCs in GM, GTEx-am and GTEx-sm respectively, maximized eGene discovery and were used for eQTL mapping in each group accordingly. GM, Greek Metabolic study. GTEx-am, GTEx-ancestry-matched. GTEx-sm, GTEx-size-matched.


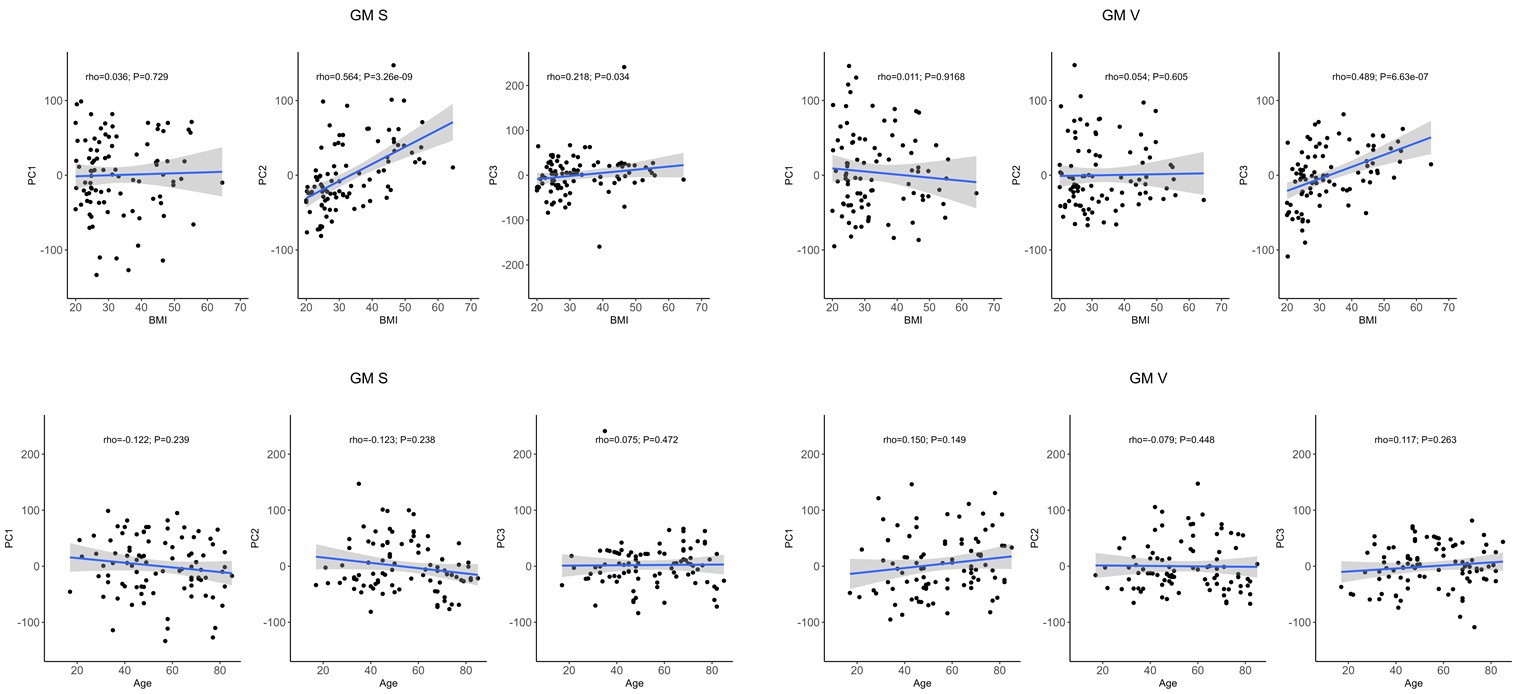


**Figure S18. Spearman’s rank correlation of first three expression principal components (PCs) with BMI (upper panel) and age (lower panel).** We assume that we removed effects of obesity status when running our differential expression analysis.


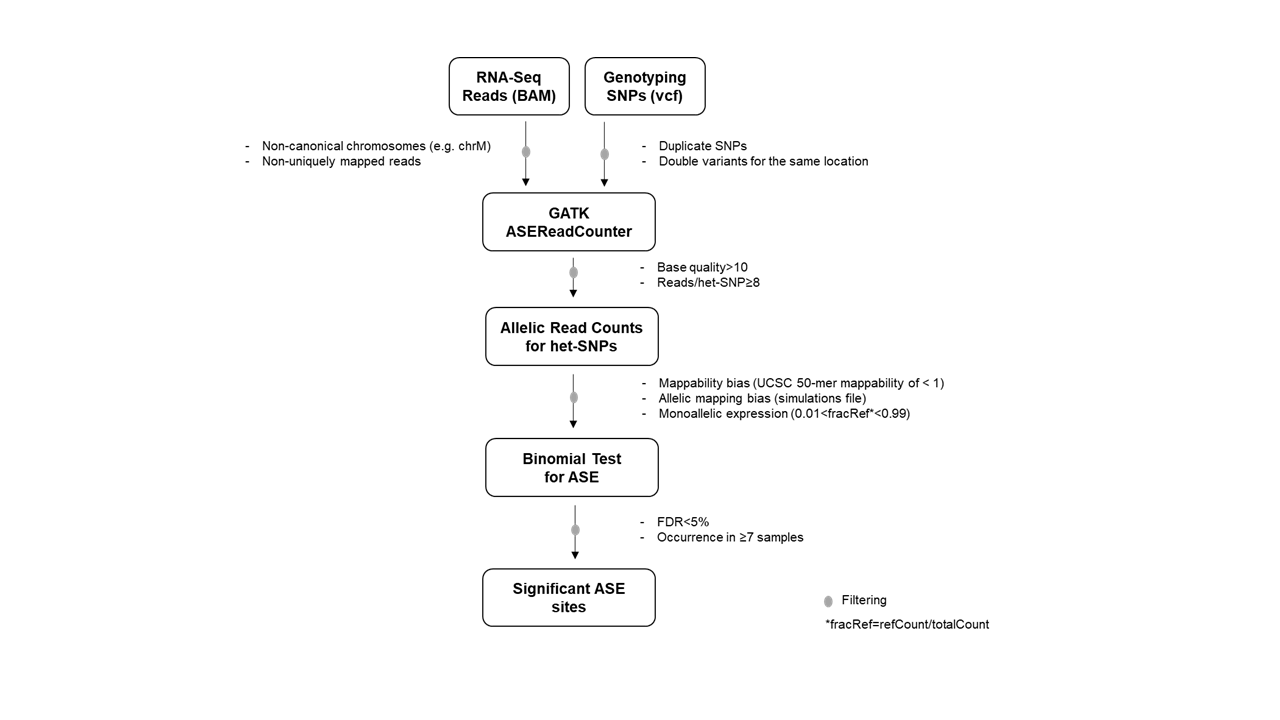


**Figure S19. Allele-specific expression (ASE) workflow in GM.**
